# Precision livestock farming and integrated molecular profiling reveal heat-stress resilience signatures in Marchigiana cattle

**DOI:** 10.3389/fvets.2026.1895058

**Published:** 2026-09-17

**Authors:** Roberta Ratto, Daniele Pietrucci, Samanta Mecocci, Marta Alonso-Hearn, Gerard Badia-Bringué, Gabriele Acuti, Francesco Renzi, Federica Gabbianelli, Stefania Bergagna, Cecilia Guasco, Davide Del Buono, Riccardo Valentini, Marco Milanesi, Piera Mazzone, Katia Cappelli, Giovanni Chillemi

**Affiliations:** 1 Department of Veterinary Medicine, University of Perugia, Perugia, Italy; 2 Department for Innovation in Biological, Agro-Food and Forest Systems (DIBAF), University of Tuscia, Viterbo, Italy; 3 Department of Animal Science, NEIKER, Basque Institute for Agricultural Research and Development, Basque Research and Technology Alliance (BRTA), Derio, Spain; 4 Nature 4.0 Benefit Company Srl, Viterbo, Italy; 5 Istituto Zooprofilattico Sperimentale del Piemonte Liguria e Valle d'Aosta, Turin, Italy; 6 Department of Animal Science, Food and Nutrition – DIANA, Università Cattolica del Sacro Cuore, Piacenza, Italy; 7 Istituto Zooprofilattico Sperimentale dell'Umbria e delle Marche “Togo Rosati” (IZSUM), Perugia, Italy; 8 Department of Experimental Medicine, University of Rome “Tor Vergata”, Rome, Italy; 9 Department of Epidemiology, Preclinical Research and Advanced Diagnostics, Bioinformatics Research Unit in Infectious Diseases, National Institute for infectious Diseases “Lazzaro Spallanzani”-IRCCS, Rome, Italy

**Keywords:** biomarkers, cattle, Cis-eQTL, gene expression regulation, heat stress, precision livestock farming, resilience, thermotolerance

## Abstract

**Introduction:**

Heat stress is an increasing challenge for cattle welfare and biological efficiency under climate change. Locally adapted and less intensively selected breeds may represent models for investigating resilience mechanisms, as they may retain adaptive traits useful for the selection of heat-resilient animals. This study characterized the response of Marchigiana cattle, a local Italian beef breed adapted to semi-extensive environments, in comparison with two cosmopolitan breeds with different productive aptitudes, Limousine and Holstein. Precision Livestock Farming, hematological profiling, and blood transcriptomics were integrated to investigate population- and breed-associated responses to heat stress (HS) under the specific farming conditions examined.

**Methods:**

IoT collars were applied only to Marchigiana cattle to record activity patterns and animal-proximal environmental variables. Blood samples from the three cattle populations were collected during summer HS and thermoneutral conditions, with sampling days selected according to the temperature-humidity index (THI).

**Results:**

Sensor-derived data in Marchigiana cattle identified distinct clusters with differences in movement intensity and animal-proximal THI dynamics during HS. Hematological analyses revealed population-specific responses: Holstein showed increased neutrophils and platelets, consistent with a stronger inflammatory profile, whereas Marchigiana exhibited higher red blood cell counts and hemoglobin under HS, suggesting better maintenance of systemic homeostasis. Transcriptomic analysis identified both shared and population-specific responses to HS. Although all populations activated core immune- and stress-related pathways, the magnitude and organization of the response differed markedly. Holstein displayed the broadest inflammatory, immunometabolic, and tissue-remodeling signature, Limousine showed an intermediate profile, whereas Marchigiana exhibited a more coordinated response characterized by immune modulation, redox protection, metabolic buffering, and less pronounced extracellular matrix remodeling. We performed expression quantitative trait loci (eQTL) mapping using gene expression data and whole-genome imputed or non-imputed genotypes. Using imputed or non-imputed genotypes, we identified 43 and 65 cis-eQTLs influencing the expression of 45 and 73 genes, respectively.

**Discussion:**

Our findings indicate that Marchigiana cattle exhibited hematological and transcriptomic profiles consistent with a potentially more balanced adaptive response to HS under the investigated conditions. However, because each breed was sampled on a different farm and direct physiological or productive measures of resilience were not included, the results do not establish an exclusively genetic breed effect or definitively demonstrate superior resilience. Integrating sensor-derived phenotypes with blood-based biomarkers may nevertheless help identify biological signatures associated with inter-individual responses. In addition, integrating transcriptomics and genomics identified cis-eQTLs that provide candidate regulatory markers for future studies of heat adaptation.

## 1 Introduction

Heat stress (HS) is generally defined as the condition in which environmental and metabolic heat loads exceed the animal's capacity to dissipate heat, thereby disrupting thermal homeostasis and triggering behavioral, physiological, and molecular responses. In cattle, HS is mainly driven by the interaction between ambient temperature and relative humidity, although additional factors such as solar radiation, air movement, housing conditions, and productive level contribute to the overall thermal burden. In the context of climate change, the increasing frequency, duration, and intensity of hot periods are making HS a major challenge for livestock production, with important consequences for animal welfare, health, and biological efficiency. Projections indicate a broader and more prolonged exposure of cattle to severe heat load in the coming decades (1–3). High-producing cattle are generally more vulnerable due to greater metabolic heat production, which elevates cortisol concentrations and induces heat shock protein synthesis, with adverse effects on immune function and inflammatory responses (4, 5). Dairy cattle are particularly vulnerable, with greater economic losses compared to beef cattle, including significant declines in milk yield and alterations in milk composition, such as reduced fat and protein content (6). However, the relevance of HS is not limited to dairy systems but also extends to beef cattle, particularly when animals are reared in outdoor or semi-extensive conditions and are directly exposed to variable environmental stressors (3, 7, 8).

Although most studies on bovine HS have focused on specialized dairy breeds, increasing attention is now being paid to beef systems and to breed-specific adaptive capacity under changing climatic conditions. Indeed, the response to HS is not uniform among breeds, since it depends on the interaction between productive aptitude, metabolic demands, management system, and genetic background. Breed and production level modulate resilience: high-yielding *Bos taurus* dairy breeds are more susceptible than tropically adapted *Bos indicus* cattle (9–11). Genetic and genomic selection, focusing on thermotolerance-associated traits such as respiration rate and rectal temperature, together with epigenetic profiling, offer promising avenues to enhance resilience without compromising productivity (7, 12).

In this perspective, locally adapted or less specialized breeds may represent valuable biological models for investigating resilience-related mechanisms, because long-term selection within specific environments may have preserved traits associated with robustness and environmental adaptability. This issue is particularly relevant for sustainable livestock systems, where the identification of animals capable of maintaining homeostasis under climatic challenge is becoming a key breeding objective (13). Previous studies in Marchigiana cattle have highlighted breed-specific genetic variants associated with immune regulation and disease resistance, including SNPs linked to resistance against bovine paratuberculosis (14) supporting the hypothesis that this native breed may retain adaptive genomic features relevant not only to disease resistance and health robustness but also to environmental resilience.

Within this framework, Marchigiana cattle are of particular interest as an Italian beef breed traditionally reared in central Italy and generally regarded as hardy and well suited to extensive or semi-extensive production systems. In the present study, Marchigiana was selected as the reference breed to investigate whether a more rustic beef genotype exhibits a different response to HS compared with two cosmopolitan breeds with distinct productive specializations: Limousine, a widely distributed beef breed, and Holstein, a high-producing dairy breed generally considered highly susceptible to heat load. This comparative design was intended to move beyond a generic description of HS and instead focus on breed-dependent biological responses, with particular attention to traits potentially associated with adaptation to heat stress.

Environmental indices such as the temperature-humidity index (THI) (15–17) remain useful for identifying periods of thermal challenge, but they do not fully capture the variability of the individual animal response. For this reason, Precision Livestock Farming (PLF) approaches are increasingly considered valuable tools for high-frequency, non-invasive phenotyping (18). In the present work, IoT collars were applied to Marchigiana cattle to continuously record movement dynamics together with animal-proximal environmental variables during HS exposure. In this context, sensor-derived data were not intended as a stand-alone diagnostic tool, but rather as a complementary phenotyping layer to be interpreted together with and transcriptomic profiles. This integrative perspective is especially relevant when the goal is to describe the HS event, moreover it may provide additional information associated with the underlying biological response.

Transcriptomic profiling is therefore a powerful tool to describe the genes and pathways activated under HS; however, it does not clarify whether differences in transcript abundance among animals are partly driven by underlying genetic variation. Expression quantitative trait loci (eQTL) mapping addresses this gap by integrating genome-wide genotypes with RNA-seq data to identify genetic variants associated with gene expression levels (19, 20). This “genetical genomics” approach, originally proposed to link DNA variation with transcript abundance, is now widely used to connect non-coding regulatory variation with complex phenotypes and to improve the biological interpretation of GWAS signals (21–23). This is particularly relevant in livestock, where many traits of economic, adaptive and welfare relevance are polygenic and are influenced by regulatory variants rather than by coding mutations alone. In cattle, recent large-scale studies such as CattleGTEx have shown that regulatory variants affecting gene expression and splicing contribute substantially to the genetic architecture of complex traits and may help prioritize candidate genes for breeding programs (24, 25). In the context of heat stress, eQTL analysis may therefore provide an additional regulatory layer to transcriptomic data, helping to distinguish genes that are merely responsive to thermal challenge from genes whose expression is under genetic control and may contribute to individual or breed-related differences in thermotolerance.

On this basis, the aim of the present study was to characterize heat-stress response in cattle through an integrated, multi-layer approach combining sensor-derived phenotyping, hematological and biochemical profiling, blood transcriptomics, and regulatory genomics. By combining these complementary sources of information, we sought to improve the biological interpretation of thermotolerance, identify molecular and physiological signatures associated with resilience or susceptibility, and provide preliminary regulatory markers potentially useful for future breeding strategies aimed at improving robustness, welfare, and heat adaptation in cattle production systems. We hypothesized that the investigated cattle populations, differing in breed, productive specialization, and farming environment, would exhibit distinct hematological and blood transcriptomic responses to naturally occurring HS. In this framework, THI was used to characterize the environmental thermal challenge, whereas the hematological and transcriptomic analyses were intended to characterize the associated systemic biological response to HS. Specifically, we expected Marchigiana cattle, traditionally reared under semi-extensive conditions, to show profiles consistent with a more balanced adaptive response than the more specialized breeds included in the study. We further hypothesized that integrating sensor-derived phenotypes in Marchigiana cattle with transcriptomic and cis-eQTL data would identify candidate biological and regulatory mechanisms associated with inter-individual variation in the response to thermal challenge.

## 2 Materials and methods

### 2.1 Ethics statement

The study was conducted in accordance with Directive 2010/63/EU and Italian Legislative Decree no. 26/2014. The studies involving animals were reviewed and approved by the Bioethics Committee of the University of Perugia (protocol number 154198, 05/05/2024).

### 2.2 Sensors

Thirty (26) female Marchigiana beef cattle located in Sant'Apollinare (Frosinone, Lazio, Italy) were enrolled in this study. All animals were reared in a semi-extensive system with access to a large outdoor paddock and pasture. Animal-related and environmental parameters relevant to health and welfare were recorded using IoT-based “AnimalTalker-Collar” neck sensors. The implemented and validated functions used in the present study measured geographical coordinates (GNSS position), environmental air temperature and relative humidity in the animal's immediate surroundings, average acceleration and standard deviation across three axes, and battery voltage. Although the collars were designed to support additional physiological measurements, including heart rate and cutaneous temperature, these sensing modules were still under technical validation at the time of the study. Therefore, only the validated environmental and accelerometric variables were included in the present analyses. They have been designed and developed by the Nature 4.0 startup (Viterbo, Latium, Italy), including both the electronic and hardware components. The collar uses LoRa technology to send data once per hour via a gateway. LoRaWAN devices send their data to an external server, where data are stored as CSV files and can be downloaded via a dedicated internet address. The sensors were kept on the animals for almost 2 months, with the 3 weeks before and after the summer heat wave (July and August) considered as the HS period. Data downloading, preprocessing and analysis was performed with Python (v 3.12) using ad hoc libraries, such as pandas (28), numpy (29), seaborn (27), matplotlib (30) and scikit-learn (26).

The tabular data were structured as follows: each row represented an hourly device measurement, while each column corresponded to a specific feature. To ensure the accuracy of the data, records corresponding to device failures (i.e., values exactly equal to zero or values with a position dilution of precision > 9,999), as well as damaged or broken devices, were removed. Subsequently, for each feature, extreme values exceeding the interquartile range were filtered out.

For each record, average acceleration, temperature and humidity were recorded. Starting from the average acceleration and the standard deviation along the three axes (x, y and z), a resultant vector (Vector Magnitude-VM) was computed as follow (28)


$$\begin{array}{l} VM=\sqrt{AC{C_{x}}^{2}+AC{C_{y}}^{2}\;+AC{C_{z}}^{2}\;} \end{array}$$

where ACC_x_, ACC_y_ and ACC_z_ are the average accelerations along the x, y and z axes, respectively. Its corresponding standard deviation (σVM) was computed with the following equation (29):


$$\begin{array}{l} \sigma VM=\sqrt{{\left(\frac{ACC_{x}}{VM}\;x\;\sigma_{x}\right)}^{2}+{\left(\frac{ACC_{y}}{VM}\;x\;\sigma_{y}\right)}^{2}\;+{\left(\frac{ACC_{z}}{VM}\;x\;\sigma_{z}\right)}^{2}\;} \end{array}$$

where ACC_x_, ACC_y_ and ACC_z_ are the average accelerations along the x, y and z axes; σx, σy and σz are the corresponding standard deviations and VM is the resultant vector magnitude. The σVM in a specific time frame reflects the intensity of the movement and may represent a proxy of movement intensity and locomotor dynamics (28). The hourly sensor-based THI was derived from the air temperature and relative humidity measured by each device, using the following formula (27):


$$\begin{array}{r} THI=\left(1.8\;x\;AT+32\right)-\left(0.55-0.55\;x\;RH\right)\;\\ x\;\left[\left(1.8\;x\;AT+32\right)-58\right] \end{array}$$

where AT is the air temperature (°C) and RH the relative humidity expressed as a fraction of the unit. The sensor-based THI is expressed in degrees Fahrenheit (°F). In addition, meteorological data (air temperature and relative humidity) were integrated from the closest weather station in Sant'Apollinare (Frosinone, Lazio, Italy), and the corresponding hourly THI was computed using the same formula described above. Then, the difference between the sensor-based THI and the weather station THI was computed and reported as ΔTHI. The rationale is to use the ΔTHI to quantify the difference between the animal-level conditions and the general climatic conditions. When animals are exposed to similar environmental conditions, ΔTHI is expected to show limited variability across individuals. In contrast, an increase of ΔTHI variability in animals under similar climatic conditions (e.g., HS conditions) could reflect differences in individual response.

After the computation of VM, σVM and ΔTHI, the records were used as input for animal phenotyping. Specifically, the dataset corresponding to the activity phase under HS conditions was reshaped into a pivot table including hourly observations of σVM and ΔTHI for each animal across the experimental period. Columns and rows with more than 50% missing values were removed. Remaining missing values were imputed using the KNNimpute algorithm (*K* = 3), based on Euclidean distance between animals (30). The imputed dataset was subsequently z-score normalized and used as input for Principal Component Analysis (PCA) to explore the distribution of samples in the reduced multidimensional space (26).

### 2.3 Blood sampling

In addition to the 30 Marchigiana female beef females with neckbased sensors, 20 Limousine beef cattle located in Viterbo (Lazio, Italy) and 20 Holstein dairy cows located in Latina (Lazio, Italy) were also enrolled in this study. All the animals were reared in a semi-extensive system with access to a very big external paddock and pasture. Blood samples were collected during both peak summer HS and the thermoneutral (TN) period. The HS period was defined as the days previous the blood sampling with a maximum THI greater than 80, indicating a moderate stress, conversely, the TN period has been defined as the period during which THI index values are below 72 units (31). Per animal, three BD Vacutainer^®^ tubes with blood clot activator (Becton Dickinson, Becton Drive, Franklin Lakes, USA), two BD Vacutainer^®^ tubes with EDTA and two BD Vacutainer^®^ tubes with lithium heparin (LH) were collected. The same standardized handling and blood-collection procedure was used during the HS and TN sampling sessions. Sampling was consistently performed in the early morning, at approximately 06:30 h, when the animals approached the feed bunk following feed delivery and entered the headlocks. Animals were then immobilized using the same procedure, and venipuncture was completed within a short and comparable interval at both sampling times. Blood samples were collected from the coccygeal vein, a minimally invasive procedure commonly adopted in cattle, by experienced veterinary personnel with specific expertise in cattle handling and blood collection.

### 2.4 Hematological and biochemical parameters

Blood samples were analyzed for hematological purpose obtaining a complete blood count [White Blood Cells (WBC), Lymphocytes (LYM), Monocytes (MONO), Neutrophils (NEU), Eosinophils (EOS), Basophils (BASO), Red Blood Cells (RBC), Mean Corpuscular Volume (MCV), Hematocrit (HCT), Mean Corpuscular Hemoglobin (MCH), Mean Corpuscular Hemoglobin Concentration (MCHC), Red Cell Distribution Width (RDW), Hemoglobin (HGB), Platelets (PLT), Mean Platelet Volume (MPV), Plateletcrit (PCT), Platelet Distribution Width (PDW)] and for biochemical purpose with hepatic and renal profile [Albumin (ALB), Creatinine (CREA), Urea (UREA), Aspartate Aminotransferase (AST), Alanine Aminotransferase (ALT), Gamma-Glutamyl Transferase (GGT), Total Protein (TP)]. Hematocrit analyses were carried out using the necessary equipment MS4 instrument in accordance with the manufacturer's instructions (Werfen, Milan, Italy), while blood samples for biochemical analysis were analyzed using an automatic photometer (BT 1,500 vet) following the manufacturer's instructions (Futurlab-Instrumentation Laboratory, Padua, Italy). The reference values used in the analyses were based on literature (32). The statistical analysis was carried out in R environment (v. 4.3.0). Selected variables were assessed for normality using the Shapiro-Wilk test and for homoscedasticity using Levene's test. Variables were transformed when necessary to meet these assumptions. For each hematological parameter, a linear model was fitted including breed, sampling condition (HS vs. TN), and their interaction as fixed effects. Analysis of variance (ANOVA) was performed to assess the significance of main effects and interactions. *Post-hoc* comparisons were performed using estimated marginal means (*emmeans* R function), and pairwise contrasts were computed to evaluate differences among breed × sampling combinations. Statistical significance was set at *p*-value adjusted for multiple testing < 0.05, and only significant pairwise comparisons were retained for interpretation.

### 2.5 RNA extraction, library preparation, and mRNA sequencing

Blood was collected in LH BD Vacutainer^®^ tubes, and buffy coat was promptly separated and frozen at −80 °C to preserve RNA integrity. The suitability of this procedure was verified by assessing the RNA Integrity Number (RIN) with the Agilent Bioanalyzer 2,100 (Agilent Technologies, Santa Clara, Ca, US), consistently with high-quality requirements for RNA-Seq, only samples with a RIN ≥ 7 were processed for downstream analysis. RNA was purified from whole blood using the Maxwell^®^ RSC simplyRNA Blood kit with the Maxwell^®^RSC Instrument. To ensure the absence of DNA contamination, residual DNA was removed by digestion with Promega DNase I (Catalog Number: Z358), following the recommended protocol. RNA samples were shipped on dry ice to IGA Technology Services Srl (Udine, Italy) for library preparation and sequencing. RNA libraries were generated using the TruSeq RNA Library Prep Kit, following the manufacturer's instructions (Illumina Inc., San Diego, CA, USA). Final libraries were quality-checked and sequenced on an Illumina NextSeq500 platform, generating 150 bp paired-end reads.

### 2.6 Bioinformatic analysis of RNA-seq data

Short reads quality was initially assessed using FastQC 0.12.0 (http://www.bioinformatics.babraham.ac.uk/projects/fastqc/), followed by the removal of low-quality bases and adapter sequences via Trim Galore version 0.6.6 (https://www.bioinformatics.babraham.ac.uk/projects/trim_galore/). The resulting high-quality reads were aligned to the *Bos taurus* reference genome (BosTau9) using the Spliced Transcripts Alignment to a Reference aligner (STAR) (33). Reads were assigned to a gene using the Ensembl Annotation 113, with expression levels quantified by counting uniquely aligned reads for each gene using the FeatureCounts function from the *R* library *Rsubread* 1.32.4 (34). Gene counts were then normalized with the mean-of-ratios method included in the *DESeq2* 1.22.2 software (35). The PCA was conducted on the normalized dataset using the top 100 most variable features and the plotPCA() function (36). Differential expression analysis was then performed to identify differentially expressed genes (DEGs) in two types of comparisons: (i) within each breed, between samples collected under HS conditions and those collected under TN conditions, the latter considered as the control group; and (ii) between breeds, within the same sampling period (TN or HS). To highlight genes more specifically affected by HS, DEGs identified in the between-breed comparison under HS conditions were filtered by removing those also detected in the TN comparison, since the latter were assumed to represent baseline breed-related differences rather than responses to HS. Result visualization was supported by volcano plots generated with the ggplot2 package. Genes were considered differentially expressed when exhibiting an absolute log2 fold change (|log2FC|) > 1.5 and an adjusted *p*-value (padj) < 0.01 after correction for multiple testing using the Benjamini–Hochberg method. Functional enrichment analysis was performed to investigate the gene ontologies (biological process, cellular components, molecular functions) and Kyoto Encyclopedia of Genes and Genomes (KEGG) pathways associated with the differentially expressed genes (DEGs) using the *R* library *ClusterProfiler* 4.14.4 (37), with *Bos taurus* gene annotation retrieved from the org.Bt.eg.db database. Enrichment results were filtered based on statistical significance, and multiple testing correction was applied using the Benjamini–Hochberg (BH) method considering a FDR-adjusted *P*-value ≤ 0.05 as significant. Additional analyses and visualization of enriched terms were carried out using the *DOSE* and *enrichplot* R packages, allowing the identification and graphical representation of the most significantly overrepresented biological processes.

### 2.7 Genotyping

Blood samples were collected into 10 ml Vacutainer EDTA tubes (BD, Franklin Lakes, USA) for genotyping. Total DNA was extracted from PB samples using the GRS Genomic DNA Kit-Blood and Cultured Cells (GRISP Research Group) according to the manufacturer's instructions (Grisp, Porto, Portugal). Purified DNA was then quantified by spectrophotometry and sent for genotyping to Agrotis srl using 100k Affymetrix AXIOM Bov 100k.

### 2.8 Imputation to Whole Genome Sequencing (WGS)

Sequence data from the Run8 of the 1,000 Bull Genomes project (38) was first phased using *Beagle 5.4*. (39) and used as reference to impute the genotypes of the study population to WGS as described in (40). SNPs with a minimum allele frequency (MAF) < 0.01 and an imputation score (R^2^) < 0.7 were excluded.

### 2.9 Cis-eQTL mapping

For *cis*-eQTL mapping, the gene expression data (normalized counts of the mapped genes) from the samples of all the cows included in the study and corresponding WGS-imputed or non-imputed genotypes were used to run *Tensor QTL*, which uses a fast permutation scheme that relies on the β-distribution to compute *P*_β_-values (41, 42). Data from both conditions (HS and TN) were included in the analysis. Breed, condition, and animal were included as covariates in the analysis to account for potential confounding effects. *P*_β_-values were corrected for multiple testing corrections with the BH method using the *R* p.adjust function.

## 3 Results

### 3.1 Sensors

We collected accelerometric data together with THI measurements obtained from both the on-farm sensor and the nearest weather station located in Sant'Apollinare (Figure 1). The difference between sensor-derived THI and weather-station-derived THI (ΔTHI) was used as an exploratory proxy of the deviation between animal-proximal thermal conditions and general farm-level environmental measurements. Sensor-derived data were used to characterize individual movement dynamics and animal-proximal thermal exposure in Marchigiana cattle during the HS period and initially comprised 31,257 records. The sensor analyses were specifically focused on the HS period in order to explore inter-individual variability under thermal challenge conditions. After quality control, 7,542 records from 19 devices were retained and used to characterize individual patterns of movement and microclimatic exposure (Figure 1). Although this represented a reduction from the raw dataset, the retained records provided adequately complete and reliable individual profiles for phenotyping purposes. To better characterize these patterns, the daily recordings were divided into two temporal phases: an activity phase (06:00–21:00) and a resting phase (22:00–05:00). This partition was defined according to the average hourly movement trend calculated across all animals during the monitoring period. The activity phase was used to identify differences in animal behavior and ΔTHI measurement, to perform following analyses.

**Figure 1:**
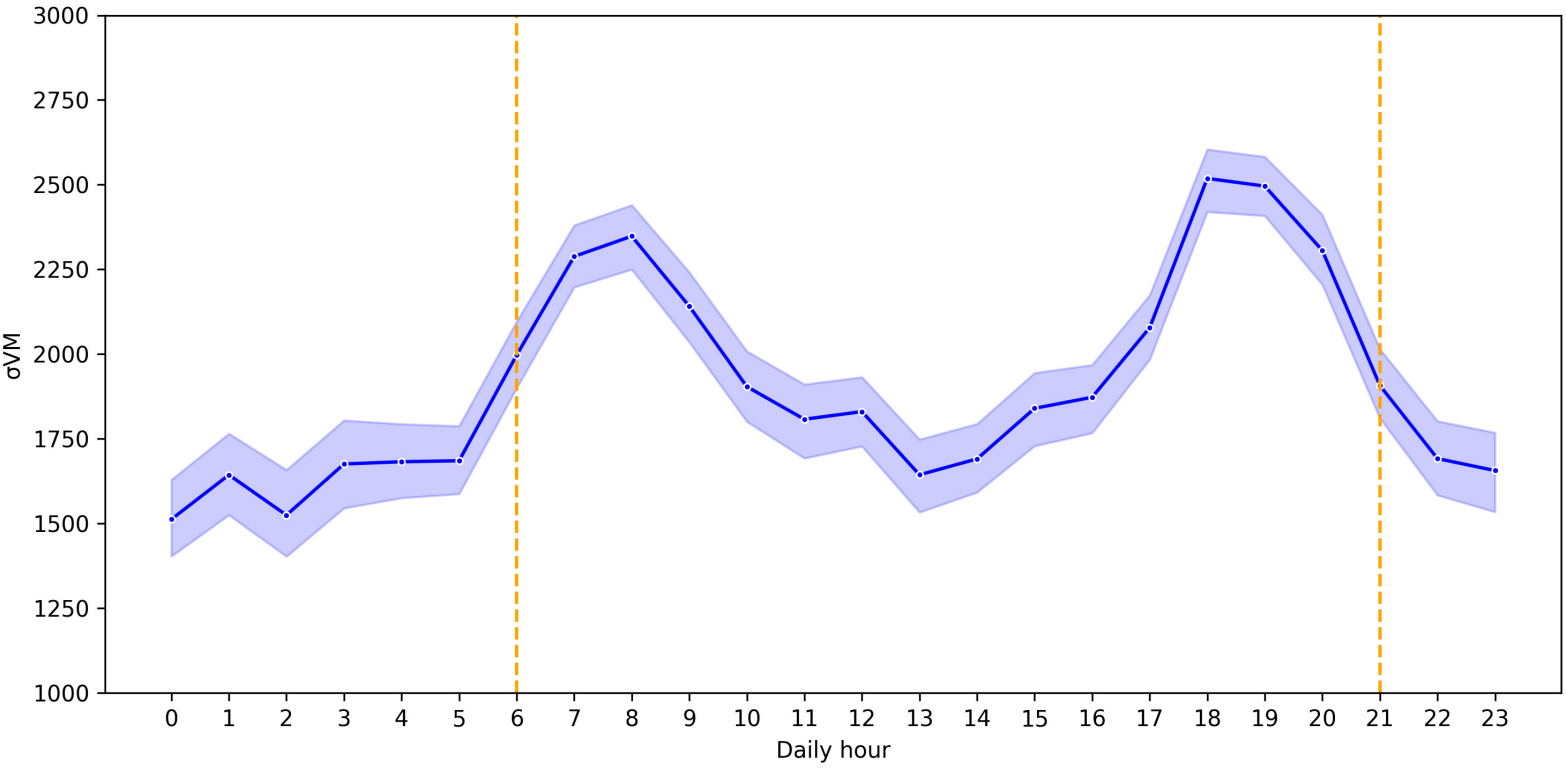
Graph showing the hourly average values of the standard deviation of resultant acceleration in Marchigiana cattle movements throughout the day.

### 3.2 Hematological and biochemical parameters

Hematological profiles of the three breeds were compared under heat stress (HS) and thermoneutral (TN) conditions (Figure 2). Marchigiana cattle showed significantly higher total white blood cell (WBC) counts under HS than under TN conditions, and WBC values were also consistently higher in Marchigiana than in the other breeds across both environmental conditions (Figure 2A). A similar trend was observed for red blood cell (RBC) count (Figure 2B), with Marchigiana displaying higher RBC values under HS than under TN, as well as higher values than both Holstein and Limousine under both conditions. In contrast, Holstein cattle exhibited significantly increased plateletcrit (PCT) (Figure 2C) and neutrophil counts (Figure 2D) under HS compared with TN, with both parameters also being higher than those recorded in the other breeds. No statistically significant differences were detected for these traits in Marchigiana or Limousine between HS and TN conditions. Both Marchigiana and Limousine showed higher eosinophil counts under HS than under TN conditions, whereas no significant variation was observed in Holstein between the two environmental conditions (Figure 2E). Hemoglobin (Hb) levels also varied according to both breed and environmental condition (Figure 2F). Overall, mean Hb values were highest in Marchigiana, intermediate in Limousine, and lowest in Holstein. However, within-breed comparisons showed that only Marchigiana displayed a significant increase in Hb concentration under HS relative to TN.

**Figure 2:**
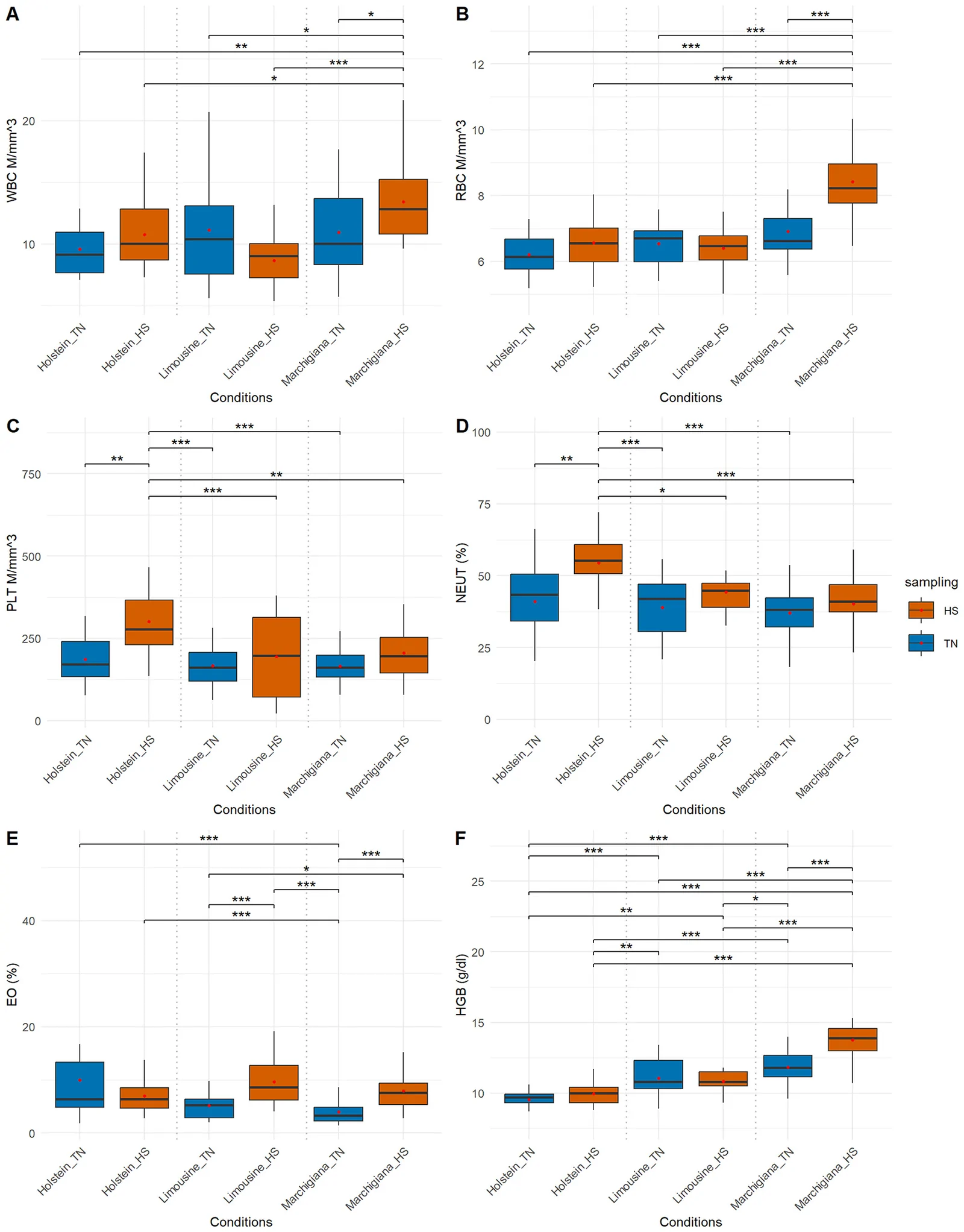
Hematological parameters evaluated in the three breeds under thermoneutral (TN) and heat stress (HS) conditions. **(A)** White blood cells (WBC); **(B)** Red blood cells (RBC); **(C)** Platelets (PLT); **(D)** Neutrophils (NEUT); **(E)** Eosinophils (EO); **(F)** Hemoglobin (HGB).

### 3.3 Transcriptomic results

#### 3.3.1 Sequencing results

The transcriptomic analysis yielded a high sequencing depth, with an average of 37.5 million reads per sample remaining after quality control and trimming procedures. An average of 36.4 million reads per sample were uniquely mapped to the *Bos taurus* reference genome. Complete sequencing stats are reported in Supplementary Table 1. An exploratory data analysis was conducted using the PCA, who showed a clear separation between HS and TN samples along the first principal component, which explained 50% of the variance in Marchigiana, 51% in Limousine, and 52% in Holstein (Figure 3).

**Figure 3:**
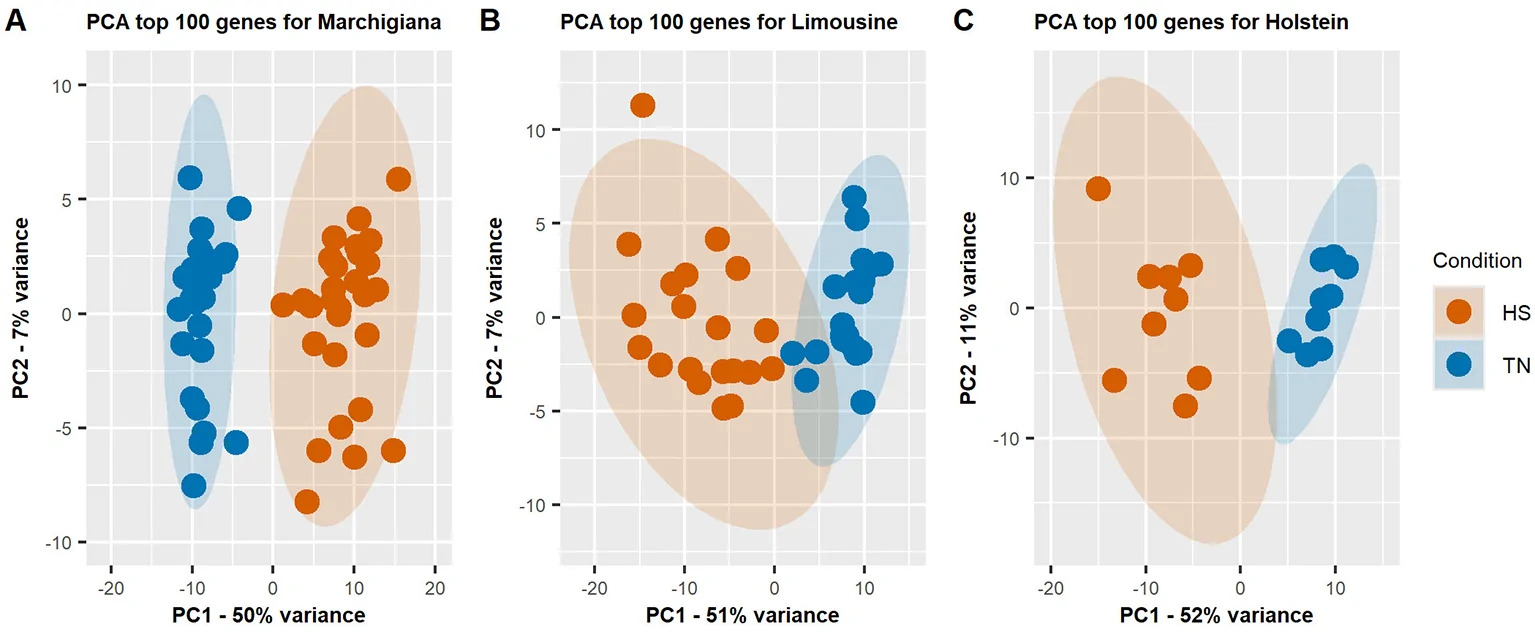
PCA top 100 genes for **(A)** Marchigiana, **(B)** Limousine and **(C)** Holstein.

#### 3.3.2 Within breed: gene expression differences between HS and TN

To investigate the transcriptional response to HS, differential gene expression analysis was performed comparing HS and TN conditions within each breed. Overall, a substantial number of DEGs were identified, highlighting breed-specific transcriptional responses to thermal stress. In Marchigiana, 199 genes were down-regulated and 620 were up-regulated under HS compared to TN. In Limousine, 104 genes were down-regulated and 447 were up-regulated, whereas in Holstein, 211 genes were down-regulated and 478 were up-regulated under HS conditions (Figure 4). The list of DEGs for each comparison is reported in Supplementary Table 2.

**Figure 4:**
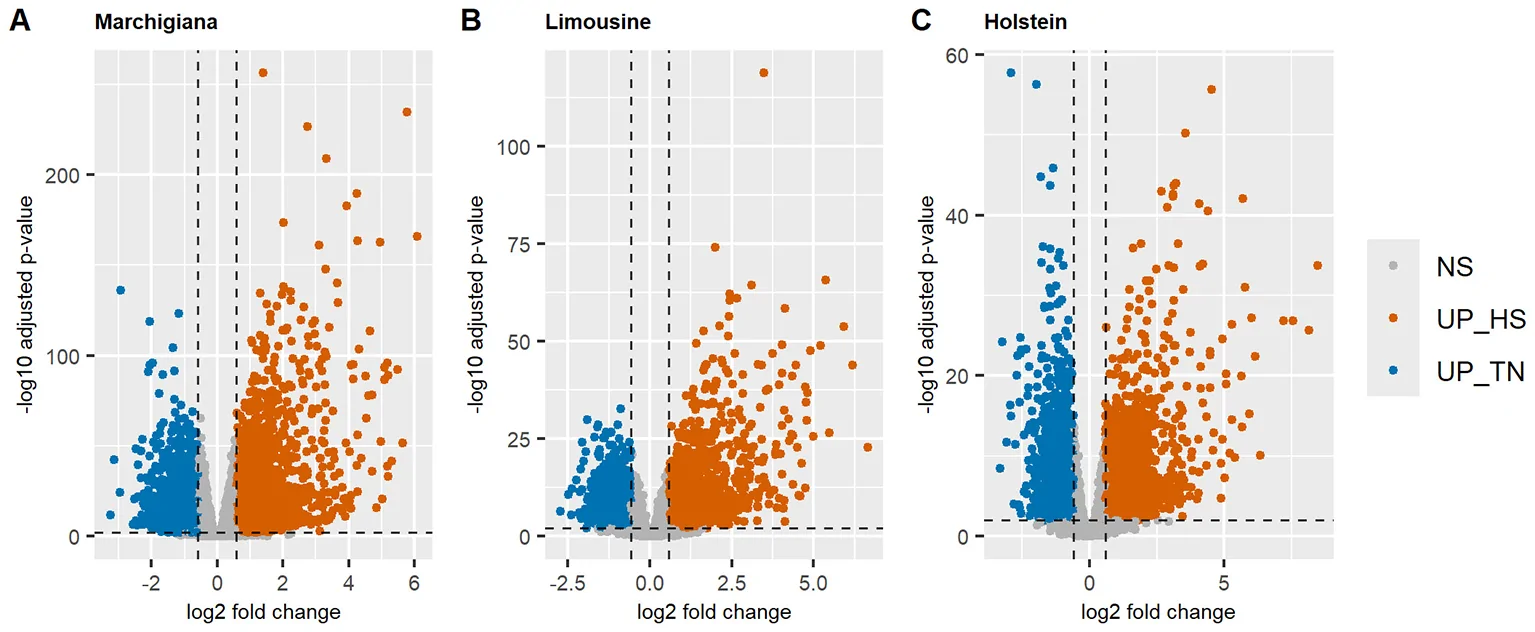
Volcano Plot of differential expression analysis HS vs TN in **(A)** Marchigiana, **(B)** Limousine and **(C)** Holstein.

The Venn diagram (Figure 5, Supplementary Table 3), based on the annotated genes of the three DEGs' list, highlighted a set of genes commonly modulated across the three breeds, suggesting the presence of a conserved core transcriptional response to HS. This shared response included genes involved in chemokine/cytokine-mediated inflammation (e.g. *CXCL8, IL10, CSF2*), early transcriptional regulation (*EGRs, FOSL1, ATF3*) and oxidative stress and energy metabolism (*NAMPT, DDIT4, PPP1R15A*). Overall, these findings indicate that the common HS response was mainly associated with inflammatory, immune, and stress-related pathways, as further supported by the subsequent functional analysis. Supplementary Table 3 summarizes all identified DEGs, indicating their presence or absence in each comparison together with the corresponding fold change (FC) values.

**Figure 5:**
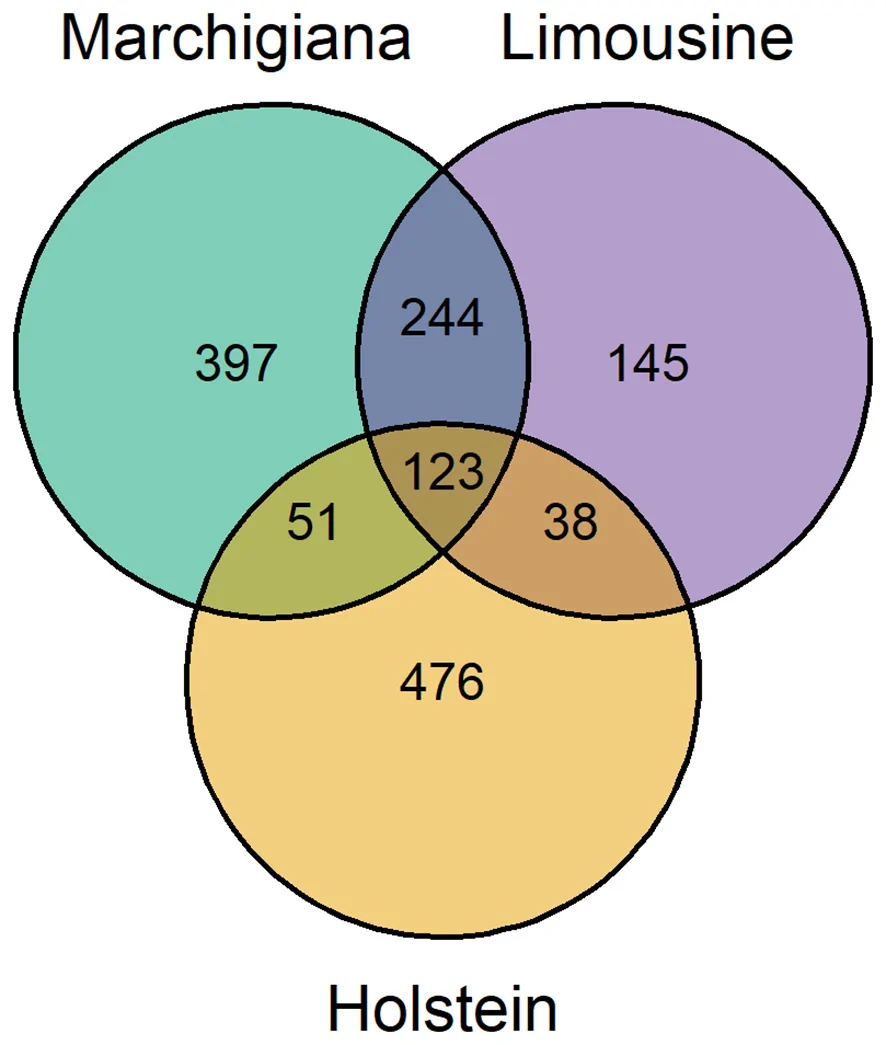
Venn diagram showing the number of unique and common DEGs between Marchigiana, Limousine and Holstein.

To gain deeper insight into the biological significance of the transcriptional changes induced by HS, a functional enrichment analysis was performed on the DEGs. Enriched Gene Ontology (GO) biological process terms and KEGG pathways were investigated to identify biological processes and pathways affected by HS within each breed (Figure 6, Supplementary Table 4). The analysis highlighted key functional responses to thermal stress, primarily associated with immune system activity, inflammation, and cellular regulation.

**Figure 6:**
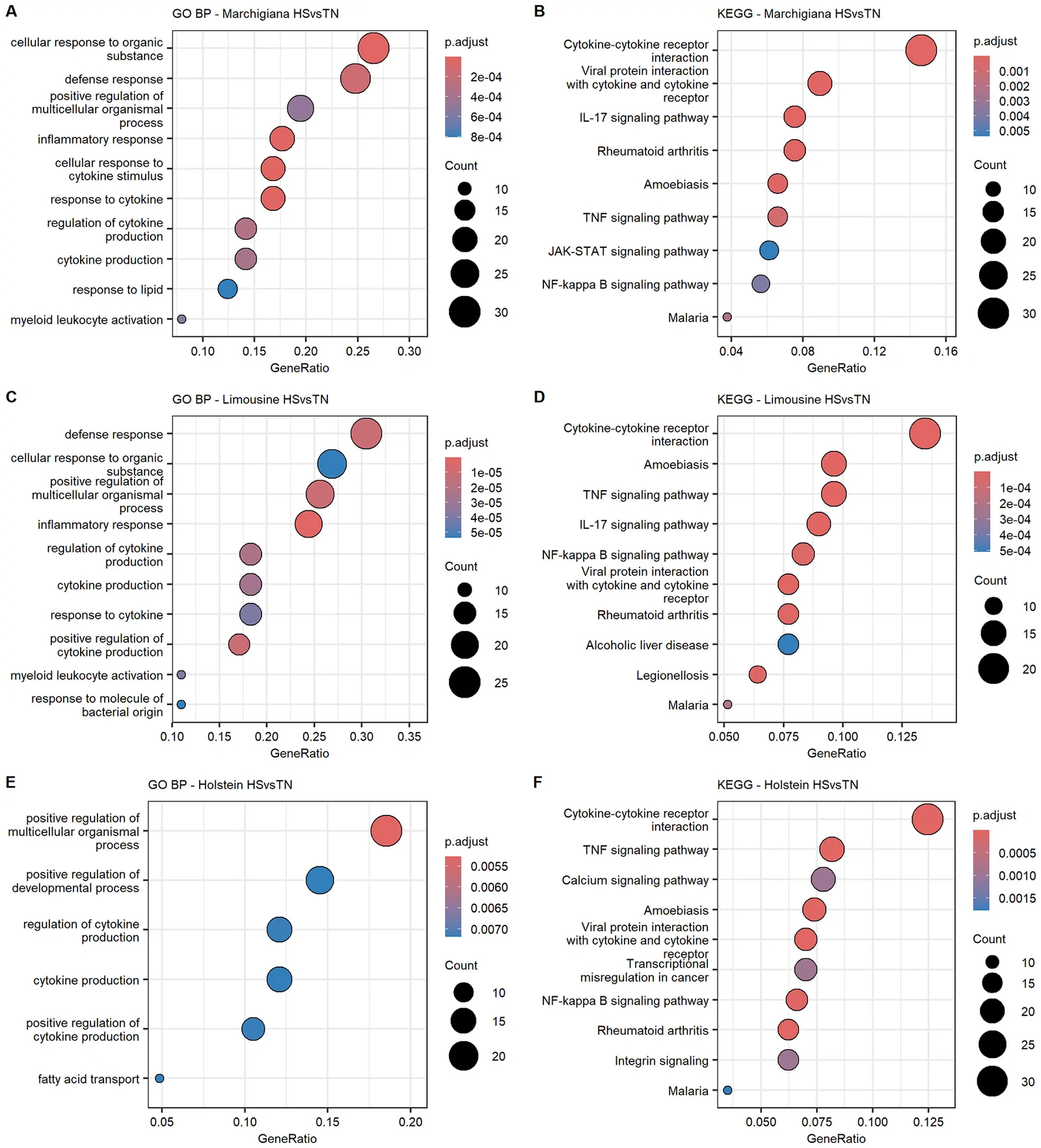
Functional Analysis for enriched Gene Ontology (GO) “biological processes” (BP) in Marchigiana **(A)**, Limousine **(C)** and Holstein **(E)** and KEEG pathways are reported for Marchigiana **(B)**, Limousine **(D)** and Holstein **(F)**.

#### 3.3.3 Between breeds: breed-specific transcriptional response to heat stress

Pairwise comparisons between breeds were conducted to better characterize breed-specific responses to HS. In particular, Marchigiana was used as the reference breed and compared with Holstein and Limousine, representing breeds with different productive aptitudes and adaptive backgrounds. This approach allowed the identification of molecular mechanisms associated with differential adaptation to HS, as well as the detection of both shared and breed-specific adaptive responses. The differential expression analysis between Marchigiana and Holstein identified 430 down-regulated and 417 up-regulated genes under TN conditions, and 557 down-regulated and 428 up-regulated genes under HS conditions (Figures 7A–B). The full list of DEGs is reported in Supplementary Table 5. After filtering HS-derived DEGs to exclude genes also differentially expressed under TN conditions, 310 genes remained uniquely up-regulated under HS and 442 were exclusively down-regulated under HS conditions, for a total of 752 HS-specific genes retained for downstream functional enrichment analyses (Supplementary Table 6). In the comparison between Marchigiana and Limousine, differential expression analysis identified 82 down-regulated and 96 up-regulated genes under TN conditions, while 230 down-regulated and 138 up-regulated genes were detected under HS (Figure 7C–D). The full list of upregulated and downregulated genes is reported in Supplementary Table 5. After filtering, 83 genes remained uniquely up-regulated and 184 uniquely down-regulated under HS conditions. These 267 genes were subsequently retained for downstream functional analyses and are summarized in Supplementary Table 6.

**Figure 7:**
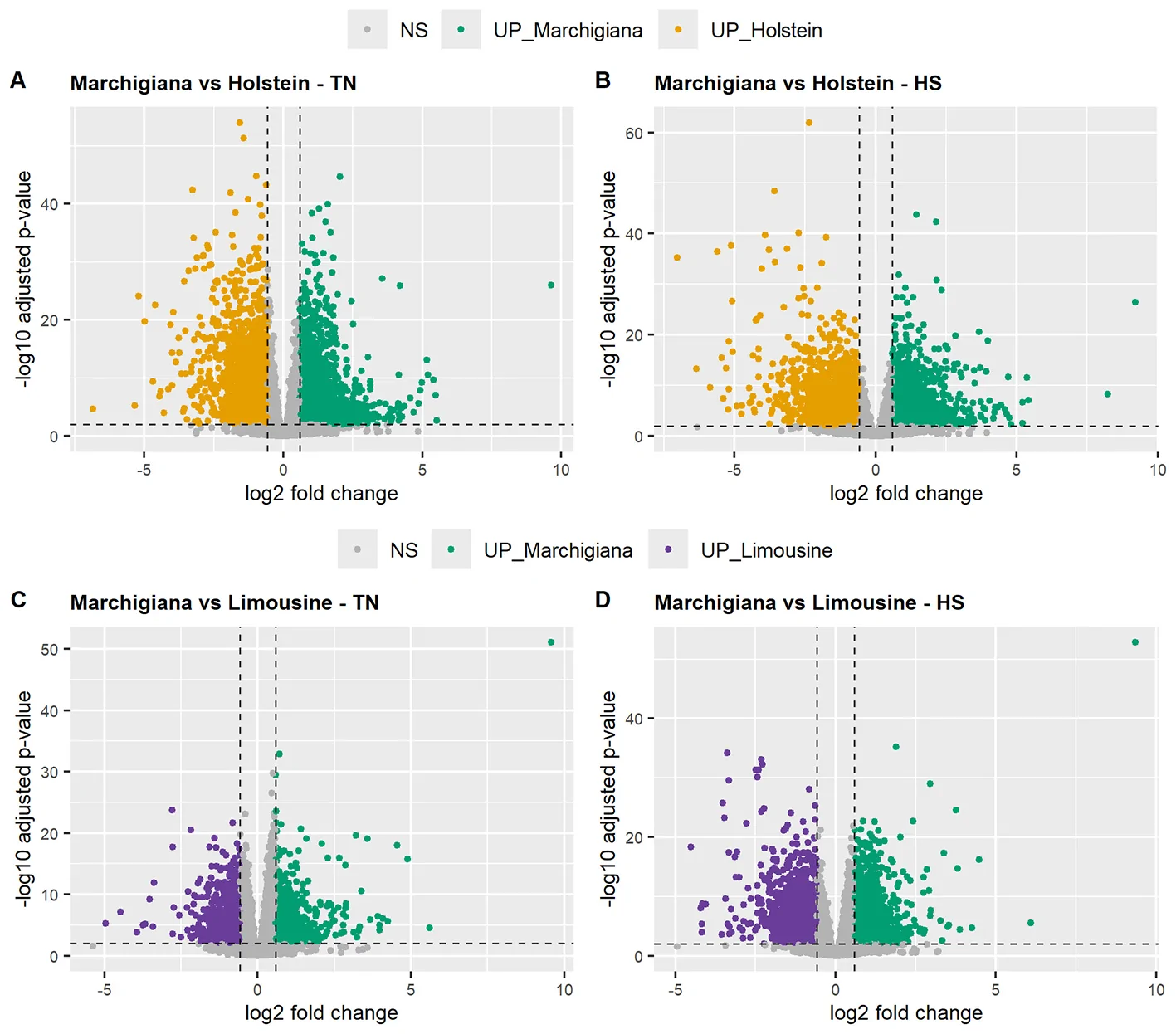
Volcano plots of differential expression analysis in Marchigiana vs Holstein and Marchigiana vs Limousine under both TN and HS conditions. **(A)** Differentially expressed genes between Marchigiana (green) and Holstein (yellow) under TN conditions. **(B)** Differentially expressed genes between Marchigiana (green) and Holstein (yellow) under HS conditions. **(C)** Differentially expressed genes between Marchigiana (green) and Limousine (purple) under TN conditions. **(D)** Differentially expressed genes between Marchigiana (green) and Limousine (purple) under HS conditions.

The use of genes specifically modulated under HS for functional enrichment analysis highlighted altered categories in terms of Biological Process (BP) only in the Marchigiana vs. Holstein comparison (Figure 8). The functional analysis of the 752 genes specifically modulated genes by HS in the Marchigiana vs. Holstein comparison (Table 1) highlighted alterations in Gene Ontology categories related to biological processes, such as “Positive regulation of developmental process” and “Positive regulation of cell differentiation” (Figure 8A). Regarding KEGG pathways, changes were observed in categories associated with immune system (Figure 8B). All functional categories are summarized in Supplementary Table 7.

**Figure 8:**
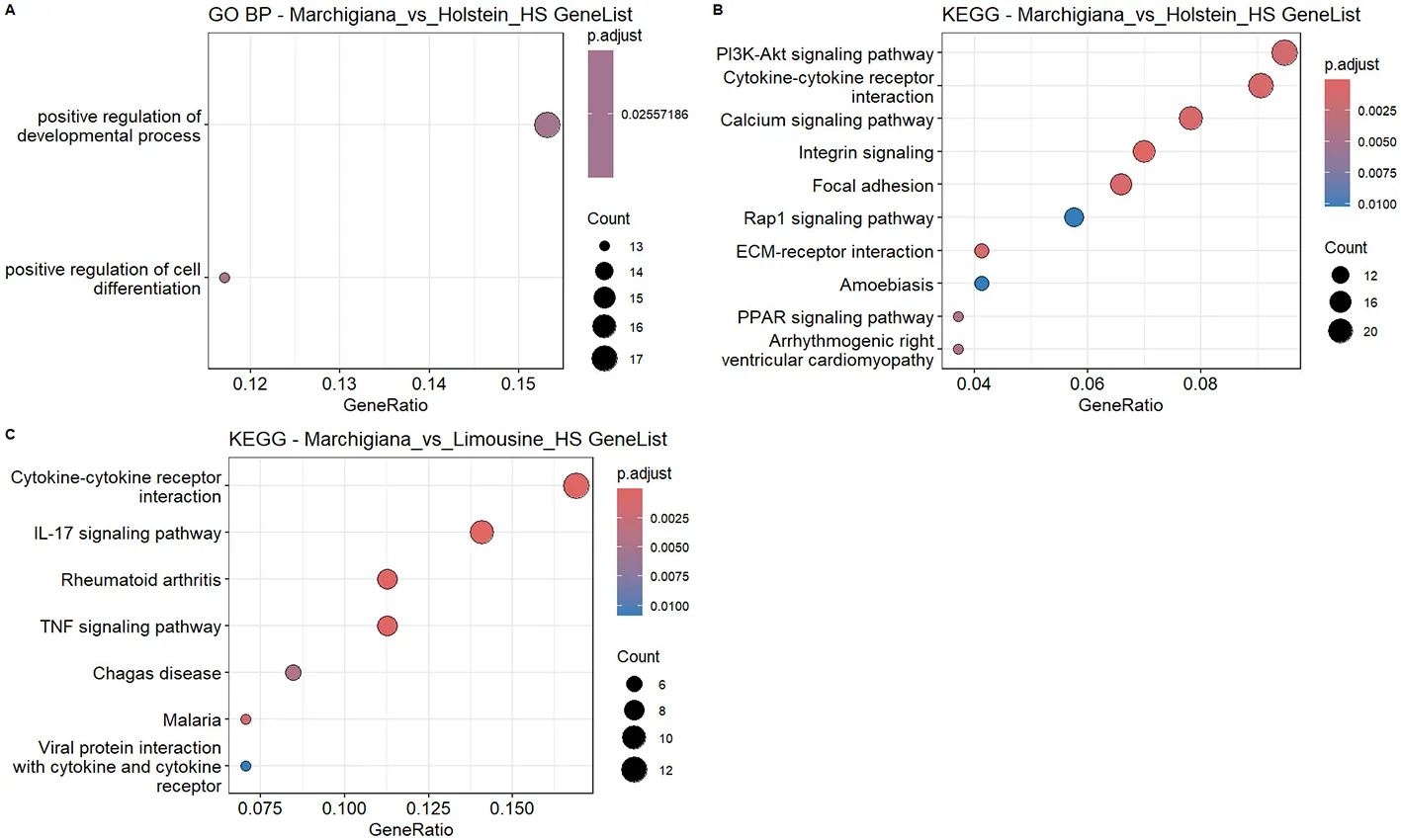
Functional enrichment of genes specifically differentially expressed under HS conditions between Marchigiana and Holstein: **(A)** Biological Processes; **(B)** KEGG pathways. Functional enrichment of genes specifically differentially expressed under HS conditions between Marchigiana and Limousine: **(C)** KEGG pathways.

**Table 1:** Specifically modulated genes by HS between breeds used for functional analysis and relative ranges of fold change.

Comparison	Regulation	SYMBOL	log2FoldChange
Marchigiana vs. Holstein	UP	***EDIL3**, GRID1, WNT5A, CDH10, LRG1, SYTL5, RASEF**, COL5A3**, TTBK1*	FC ≥ 3
		*DCT, ZC4H2, KCNH8, **CALCRL**, WSCD1, FGF13, BEX2, SLC6A1, TFF2, KCTD19, KCNQ4, SAMD10, TRIM47, MMP15, GAS1, SLC6A16, **PRKAA2**, MMRN1, TPC3, ZNF84, DNAI1, LYPD3, ATRNL1, PLPPR5, MCF2, PBX1, LHFPL7, SGSM1, POGLUT3, NEXMIF, MYO5C, SSPO, MAB21L3, PHACTR3, SLC22A7, LARP6, KIF6, KLRJ1, **DTX1**, TUB, PCDH9, CPNE8, TTLL7, GNA11, ITGA3, AR, DNAI4, CRIP3, TMEM213, PGBD5, OSCP1, TLR5, FGF13, PCDH19, DSB, ZNF33B, GDF11*	2 ≤ FC < 3
		*MAMDC2, EVC2, **SPARC**, SRMS, ME1, EGR4, TMEM205, WNT8B, BAHCC1, CACNA1I, ADGRG1, PEG10, XPNPEP2, AMOT, FOXQ1, C1H3orf52, SNX33, CHRM3, TGFB2, B3GLCT, CORO2B, FLRT1, PRKCG, DKKL1, **BTLA**, JAM2, RNASE13, BEND7, GCA, WC1, ANKS6, SLC22A20, ZDHHC1, PGA5, TARP, JHY, ABCC5, U6, RFX2, DNAH9, ENHO, NUGGC, HORMAD1, CD163L1, RTN4RL1, SFXN5, KCNJ1, EXOC3L4, SLC24A2, ADCY8, TRPM1, BACE2, CRPPA, GHR, ZNF618, TMTC1, MOGAT1, SCRN1, GREB1, CABCOCO1, SAMD4A, MINDY4, GABBR1, COL18A1, BSX, TRIM46, SCD5, SMAD6, CYB5R2, MBOAT2, SH3D19, CACNB3, SYT7, ARMH4, FGF11, CFAP91, PKP2, EFHC2, **SIGLEC11**, SPRY3, ANKUB1, MANSC1, ARHGEF10, FSD1, FAM171B, KLHL32, AMPH, IFT27, TLR7, TTC22, **IGFBP4**, RASAL1, ZNF554, WNK3, DNAAF9, KIAA1549, IL17RD, RPGRIP1L, PLCL1, SH3GL3, CYP2J30, CCDC9B, FAT4, PROKR2, ADGRL1, NLRP13, NID2, CPXM2, SNTG2, DAB2, FBXO16, ZNF318, **THEMIS**, HVCN1, CYP27A1, **ALAS2**, ECHDC3, MBNL3, ZMYND12, SERPINI1, AOX4, ASRGL1, MAP3K9, IQSEC2, SEMA6C, SLC34A3, GAB1, **GPIHBP1**, TBC1D21, PTER, SOX4, PRG3, ENDOU, L1CAM, SAC3D1, ADAMDEC1, **RNF128**, TRPC6*	1.5 ≤ FC < 2
	DOWN	*CYP3A74, OLFM1, ANKRD65, DENND2A, TSFM, PHLPP1, CELF3, MSR1, FAM72A, **IL1RN**, CATIP, TBC1D9, ZDHHC2, EDNRB, JAG1, LPAR5, RAB7B, TMEM158, BCL2A1, **VEGFC**, HSD3B7, EEF1AKMT3, TRAF1, SLC7A10, CCL4, LHFPL2, SLC11A1, FAM178B, BASP1, CYP27B1, P2RY6, C9, FCRL3, **ISG15**, NAB2, DGKH, SCNN1D, NEIL3, MUC2, NMUR2, FAP, LGI4, CTTN, FHL3, ADAMTSL2, PPARD, AK1, **SOCS3**, C1QL1, **ACSL4**, FSCN1, FLNC, SCHIP1, CACNA1F, ABTB2, PXYLP1, LOXL1, bta-mir-2388, NLRP8, **ACOD1**, NPNT, ASPRV1, IL27, LRRC71, PLEKHA4, RXFP4, SIPA1L2, SCG3, ATP13A3, TECTB, MS4A3, SNORA70, LRRC32, AK4, TRPV3, LRRC25, **VEGFA**, MYO10, TREH, MARCHF3, CCDC24, ADAMTS12, SEC24D, 5S_rRNA, VGF, CDH20, **TREM1**, GLIS3, MYRF, PDGFRB, CPNE5, **COL4A1**, DCBLD1, LAMC3, CD200R1, ST6GALNAC6, VDR, bta-mir-222, SEMA5B, CKB, **OASL**, PEAK3, DPF1, DPYS, CGNL1, CAMKK1, FAM20A, IQCK, GSTM3, CD68, KCNMA1, **ABCG1**, ZMYND10, ADORA2A, **VASH1**, TEX101, **LAMA5**, ADAMTS4, CHSY1, FMNL3, ETV5, CDH4, OSMR, CLEC10A, SH3PXD2B, PDE9A, CRABP2, DOT1L, IGFBP1, MMP14, SCIN, MGAM*	−2 < FC ≤ −1.5
		*MN1, bta-mir-221, SORCS2, CLEC4A, **APOE**, BATF3, PPP2R2C, GFPT2, MYMK, PBX4, TRIM72, ITGB5, BCAR3, GLP1R, CTNND2, EDN1, PLXNA1, SPRY4, TTLL10, ANKRD37, S100A2, **CLEC5A**, ADM, ADAMTS1, **VCAN**, SPRED2, STOML3, MGAM2, MT2A, TNFRSF11A, ETV3L, KDR, CYP21A1P, LTB4R2, SLC44A3, **VCAM1**, CDA, ITGB8, CFB, SNAP25, SCARA5, VEPH1, AHRR, KCNQ1, HIP1, ADGRG3, RIN2, MEDAG, CELSR3, NGF, PPP1R3C, OAF, IL18BP, **TNIP3**, TIFAB, SLAMF8, **IL1RAP**, LIPN, GPR153, OR2AT2, CNN3, SLC5A1, **FLT1**, TNFAIP8L3, TNFRSF18, **LPL**, SLC39A8, **CMKLR1**, ATP13A4, ONECUT2, CXCL10, JUP, HIVEP3, SRC, CD300E, **GDF15**, U2, SLC2A10, TMEM151B, PRDM9, UPB1, C19H17orf67, ACTN2, **LIPG**, GATA2, GJA1, BMP1, **HMOX1**, RBPJL, DMXL2, HTR7, **ABCA1**, COX4I2, LYPD5, DCSTAMP, CAPN14, **WWTR1**, FOXF1, FFAR2, GSDME, IL36A, AUTS2, SERPINB2, OCSTAMP, **FABP5**, FGF1, GPRC5A, RAPH1*	−3 < FC ≤ −2
		*HTRA1, MAMLD1, TIMP1, CAMK1G, SERPINB8, SNORA70, EPOP, CCL24, GZMB, KRTAP11-1, **CXCL13**, **CSF3**, GAL, CCDC80, **MMP1**, SLC22A3, CLDN4, IGFBP6, MERTK, LTB4R, PTGIR, LAMB3, F11, SGMS2, MMRN2, RGL1, HCRTR1, **FCGR1A**, RAB3IL1, YAP1, KCNH4, GPC1, IL9, **CCL20**, LVRN, **FLT4**, SAA3, **CX3CL1**, EHF, MMP12, RAB15, **CH25H**, **CCL8**, ADAMTS8, CLDN1, **CD36**, ARL14EPL, **CCL22**, **PDPN**, SPP1*	FC ≤ −3
Marchigiana vs Limousine	UP	*-*	FC ≥ 3
		***KLF15**, KCNH8, **OSGIN1**, KLHL33, **WNT5A**, TMPRSS4, **WWC1**, RBM24, LARP6, OXT, **OSM**, MACC1*	2 ≤ FC < 3
		*GRID1, SCN4B, **DCXR**, **HEY2**, **LRG1**, PROKR2, ANKS4B, ATP4B, CRABP1, SLC52A3, FOXQ1, **SIGLEC1**, **GATA4**, HIC1, RTL9, TFAP2C, PEAR1, ZNF503, **CCL24**, FOS, NINJ2, MSMB, **JUP**, PAQR5, TEKT3, **CTLA4**, KCTD19, PEG10, **MSRB3**, **KIR3DL2**, GULO, **CYB5R2**, TMEM213, **PPP1R3B**, CEMIP, PDE11A, **PLIN1**, **IL21**, OSCP1, MANSC1*	1.5 ≤ FC < 2
	DOWN	*CPLX3, AKAP12, NGEF, CDKL4, **ADM**, RNU6ATAC39P, GZMB, **RARRES2**, VSTM2B, MAP4K3, APCDD1L, U6, NPNT, PPP1R1B, SOX5, TMEM45B, MAPK10, RHBDL1, COX6A2, TRPM5, ZBTB32, FOXO6, bta-mir-6530, VEPH1, COCH, CD36, LMCD1, RNF222, HBA, **MMP1**, SNORA79, bta-mir-12027, SERPINB2, CYP21A1P, SNRPD1, U6, SMC1B, bta-mir-33a, SEMA5B, **NOS2**, NKX6-2, DYNLL1, EPHX4, **PDK4**, RNase_MRP, CDC42EP1, OR52B3, ABRA, bta-mir-11999, bta-let-7a-3, **CXCL3**, INSYN1, TSPAN1, U6, SHISA3, **PTGS2**, **TFPI2***	−2 < FC ≤ −1.5
		*SNORA70, ADAMTS1, CLDN8, CPA3, ATP13A4, KAZN, CFB, TRPC2, PPP2R2C, **IL9**, bta-mir-210, **CCL2**, ADAMTS13, U2, TRDN, **CSF3**, PALLD, KDR, **IL36A**, ATP12A, **CSF2**, **PDPN**, NGF, GATA2, LIPN, STOML3*	−3 < FC ≤ −2
		*ANKEF1, CLDN1, **IL6**, EEF1A2, NKX3-2, EHF, **CCL20***	FC ≤ −3

Particularly interesting genes are highlighted in bold and commented in discussion.

For the specifically regulated genes under HS conditions between Marchigiana and Limousine (Table 1), no significantly altered biological processes were identified, but changes in KEGG pathways were observed, related to immune system signaling (Figure 8C). No differences were observed between Marchigiana and Holstein in terms of KEGG pathways.

### 3.4 Cis-eQTL MAPPING

The obtained genotypes comprised a total of 76,506 variants with a call rate greater than 95%. In total, 4,130,630 variants per animal, including small insertions and deletions, were obtained after WGS imputation with an imputation score (*R*^2^ > 0.7). SNPs with a minimum allele frequency (MAF < 0.01) were excluded. After applying these quality parameters, 3,880,663 SNPs passed the filters and were used for the cis-eQTL mapping.

Significant associations (FDR ≤ 0.1) between cis-eQTL located within 1 Mb upstream of a TSS and normalized gene counts were detected using WGS-imputed and non-imputed genotypes. The cis-eQTL identified within 1 Mb upstream of a TSS that are associated with changes in gene expression levels are presented in Figure 9. Using the WGS-imputed genotypes, we identified 43 cis-eQTL (Figure 9A) and 65 cis-eQTLs were associated with expression changes of gene expression using non-imputed genotypes (Figure 9B). Most of the identified cis-eQTLs we located in intronic regions in the WGS-imputed (59%) and non-imputed (64%) genotypes (Figures 9C and D). These identified cis-eQTL and their target genes are presented in Supplementary Table 8.

**Figure 9:**
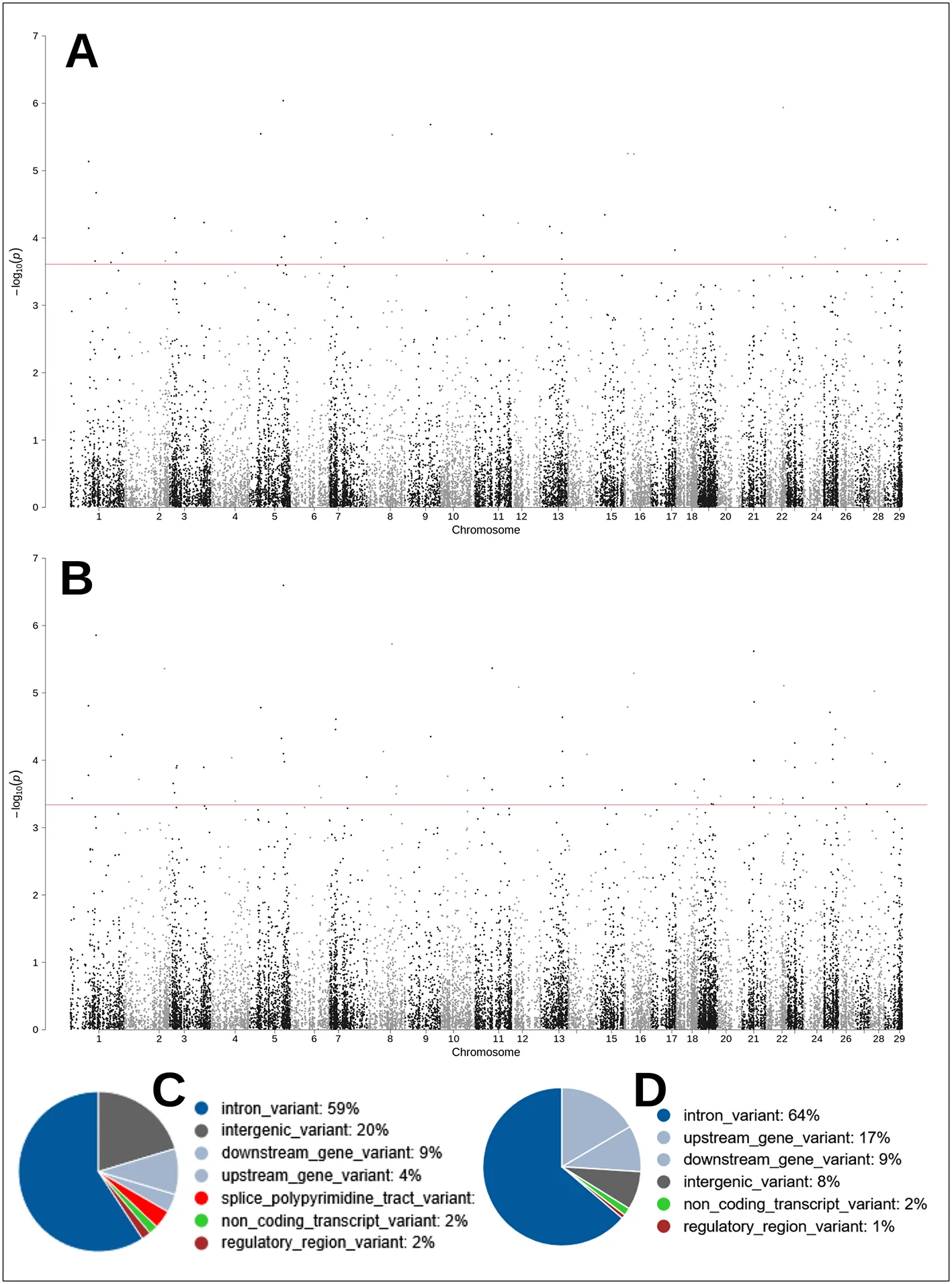
Manhattan plots of cis-eQTL mapping results. Cis-eQTL identified within 1 Mb upstream of a TSS using WGS-imputed **(A)** and non-imputed **(B)** genotypes. The plot shows in the Y axis the –log10 (Pβ-values) of each SNP and in the X axis the chromosome where each cis-eQTL is located. Each dot represents a SNP along the Bos taurus genome. The dotted lines represent the Pβ-values that correspond to a FDR equal to 0.05. The charts show the genomic distributions of the cis-eQTL identified within 1 Mb upstream of a TSS using WGS-imputed **(C)** and non-imputed **(D)** genotypes according to the Ensembl Variant Effector Predictor (VEP).

To directly integrate differential-expression and cis-eQTL results, we identified six transcripts that were both differentially expressed between HS and TN conditions and associated with a cis-eQTL at the exploratory threshold adopted in this study (FDR ≤ 0.10; Supplementary Table 9). These comprised four protein-coding genes—NRL in Marchigiana cattle and CHRND, GPRC5B, and KRTAP11-1 in Holstein cattle—and two currently unnamed long non-coding RNA (lncRNA) genes, ENSBTAG00000060972 in Limousine cattle and ENSBTAG00000063123 in Holstein cattle. The associations involving CHRND, GPRC5B, and ENSBTAG00000060972 also met the more stringent FDR ≤ 0.05 threshold.

### 3.5 Sensors derived clustering and transcriptomic analysis in marchigiana cattle

Based on the hourly profiles of movement intensity and ΔTHI, PCA clustering identified three groups of animals. Cluster 1 included eight animals, Cluster 2 included five animals, and Cluster 3 included three animals. When the same animals were projected into the PCA space, using the *K*-means cluster membership as grouping information, Cluster 3 showed a clearer separation from the other two clusters, whereas Clusters 1 and 2 were more largely overlapping (Supplementary Figure 1). Inspection of PCA loadings suggested that PC1 was mainly associated with ΔTHI-related variation, while PC2 was more strongly influenced by movement-related variables. The comparison of hourly ΔTHI profiles among clusters showed that animals belonging to Cluster 3 displayed higher values during the early hours of the day, particularly between 06:00 and 08:00, compared with animals from Clusters 1 and 2. This pattern tended to reverse between 09:00 and 16:00, whereas only minor differences were observed after 17:00 (Supplementary Figure 2A). Movement intensity showed greater variability than ΔTHI; however, animals in Cluster 3 generally displayed higher movement values than those in Clusters 1 and 2, while Cluster 1 tended to show higher movement than Cluster 2 (Supplementary Figure 2B). Overall, these results indicate that Cluster 3 represented a small subgroup of animals characterized by partially distinct sensor-derived movement and ΔTHI profiles during HS exposure.

To evaluate whether the sensor-derived phenotypic classification was reflected at the transcriptomic level, RNA-seq data from Marchigiana animals were re-analyzed according to the three clusters identified by K-means. PCA based on transcriptomic profiles showed that the three animals assigned to Cluster 3 tended to group closer to each other and to be partially separated from the remaining animals. Conversely, animals belonging to Clusters 1 and 2 showed a more overlapping distribution, suggesting a lower degree of transcriptomic differentiation between these two groups (Supplementary Figure 3).

Differential expression analysis was then performed using the sensor-derived clusters as experimental groups. Given that all animals belonged to the same breed and were sampled during the same environmental period, an exploration differential expression analysis was performed using a less stringent statistical threshold, appropriate for hypothesis-generating analyses in small subgroups (p-adj < = 0.1 and |log2(FC)| > = 0.58). This analysis identified 10 differentially expressed genes in the comparison between Cluster 1 and 2, whereas a much larger number of genes was detected in comparisons involving Cluster 3, with 1,772 DEG in Cluster 1 vs. Cluster 3 and 1,369 DEG in Cluster 2 vs. 3 (Supplementary Figure 4, Supplementary Table 7). Functional enrichment analysis highlighted general KEGG pathways related to translation and cellular activity, together with signals involving MAPK-related pathways in the comparisons including Cluster 3 (Supplementary Figure 5, Supplementary Table 8). Although these results should be interpreted with caution due to the limited number of animals, they suggest that the sensor-defined Cluster 3 may correspond to a subgroup with both behavioral and transcriptomic differences under HS conditions.

## 4 Discussion

In this study, an integrated assessment of stress and welfare indicators was carried out through an integrative phenotyping approach, combining the use of biosensors and RNA-Seq analysis within the framework of Precision Livestock Farming (PLF). A total of 30 Marchigiana female beef cattle with neck-based sensors located in Sant'Apollinare (Frosinone, Lazio, Italy), 20 Limousine beef cattle located in Viterbo (Lazio, Italy) and 20 Holstein dairy cows located in Latina (Lazio, Italy) were enrolled. We started with the application of IoT Nature4.0's biosensors to the Marchigiana beef cattle, we proceeded with the sampling of all the animals enrolled for hematological and biochemical analysis and for RNA-Seq. We aimed to assess cattle welfare in HS conditions in order to identify biomarkers potentially associated with adaptive responses to HS that could be useful for breeding selection in a sustainable farming system. The integration of sensor-derived phenotypes framework provided an exploratory approach to characterize inter-individual variability under HS conditions in Marchigiana cattle. Pertaining to the sensors analysis, the use of wearable IoT devices represents an innovative approach to monitor beef cattle under semi-extensive management systems. In this study, the sensors were able to provide continuous information on both animal-related and environmental parameters.

Regarding the comparative hematological and biochemical analyses across Marchigiana, Limousine, and Holstein, they provide important insights into the physiological responses of different breeds to seasonal variations and HS conditions. The rise in total white blood cells (WBCs) observed in Marchigiana cattle may reflect an adaptive immune response to environmental stressors, including elevated temperature and humidity. In contrast, Holstein cows exhibited a marked increase in neutrophils, which suggests a stronger inflammatory response. This finding is consistent with previous studies, which had already observed that Holstein cows had higher WBCs under HS (43, 44). This breed-related difference could be due to the higher metabolic demands of dairy cattle, making Holsteins potentially more susceptible to heat-linked oxidative and inflammatory processes. The eosinophilia detected in both Marchigiana and Limousine cattle during HS is consistent with seasonal parasitic burdens, which are known to increase under warm and humid conditions (45–47). This highlights the importance of integrated parasite management in semi-extensive systems, particularly during peak summer periods. Red blood cell (RBC) count and hemoglobin concentration are elevated in Marchigiana cattle, which may represent a compensatory mechanism to enhance oxygen delivery under HS conditions, when respiratory and circulatory adjustments are crucial for maintaining homeostasis. On the other hand, Holsteins showed thrombocytosis and an increased plateletcrit (PCT), which further supports the hypothesis of a heightened inflammatory state in this breed during HS exposure (48, 49).

Regarding the RNA sequencing results, we performed two complementary differential expression analyses, considering the three different breeds (Marchigiana, Limousine and Holstein) and the two different seasons (HS and TN). First, we evaluated the effect of HS within each breed (HS vs. TN). Notably, in all three breeds, the number of up-regulated genes exceeded that of down-regulated genes under HS. A similar predominance of transcriptional activation has been reported in other bovine heat-stress transcriptomic studies, including Holstein calves and lactating Holstein cows, suggesting that HS often induces an active adaptive response rather than a mainly suppressive transcriptional program (50–52). A plausible explanation is that HS requires the rapid induction of protective pathways involved in cellular homeostasis, including stress signaling, immune and inflammatory responses, oxidative-stress control, and metabolic adjustment. However, this pattern should not be considered universal, the relative prevalence of up- or down-regulated genes may depend on breed, tissue, physiological status, and heat-stress model. Despite some breed-specific differences in the genes involved, all three breeds showed enrichment of KEGG pathways and GO terms mainly related to immune function inflammatory responses. The enrichment results highlight substantial differences in genes involved in the regulation of the immune system and energy metabolism (Figure 6). These findings are in line with the evidence that thermotolerance is strongly interconnected with both innate and adaptive immunity, as well as with energy metabolism (49, 53–57).

Among the shared responses, genes related to Toll-like receptor signaling and cytokine regulation emerged as particularly relevant. HS has been associated with increased circulating lipopolysaccharides (LPS) due to impaired gut integrity, which may activate acute-phase and inflammatory responses through Toll-like receptors, particularly the TLR2/4 pathway (53). Consistently, in our dataset, *TLR4* was upregulated in Marchigiana and Limousine under HS, whereas in Holstein it showed a weaker response. Since *TLR4* has been described as an important mediator of long-term immune adaptation to HS in cattle, especially in more thermotolerant backgrounds, this pattern may suggest a more marked activation of this pathway in the two beef breeds (58). A similar trend was observed for *TLR10*, a receptor with anti-inflammatory functions, which was downregulated in all breeds, although more markedly in Marchigiana and Limousine (59). Cytokine-related genes further supported the activation of inflammatory pathways across breeds. In particular, *IL6* was upregulated in all breeds, with a stronger fold change in Holstein than in Marchigiana and an intermediate response in Limousine, in agreement with previous observations showing higher *IL6* levels in more heat-sensitive cattle (60, 61). Notably, the ribonuclease *ZC3H12A*, which promotes *IL6* mRNA degradation (62), was significantly upregulated only in Marchigiana and Limousine, suggesting a possible breed-related difference in the post-transcriptional control of inflammation. Likewise, *IL1B* was upregulated under HS in Limousine and Holstein, while in Marchigiana the increase was more limited, confirming some variability among breeds also for this pro-inflammatory mediator (63–65). In contrast, the anti-inflammatory cytokine *IL10* was differentially expressed in all three breeds, with the highest fold change in Marchigiana.

Overall, these results indicate that HS induced a common immune-inflammatory transcriptional response across the three cattle populations, but with quantitative differences in key regulatory genes. We then compared the populations within the HS condition to identify transcriptional features associated with their different response patterns. These comparisons revealed distinct transcriptional programs differing in inflammatory intensity, metabolic cost, and apparent capacity to preserve homeostasis. However, because each breed was represented by animals from a different farm, these contrasts should be interpreted as population- or breed-associated patterns under the specific farming environments investigated, rather than as differences attributable exclusively to genetic background. Within this framework, Holstein displayed the broadest and potentially most energetically demanding response, Limousine an intermediate but clearly reactive profile, and Marchigiana a more regulated and homeostasis-oriented pattern. The hematological data were consistent with this interpretation: Holstein showed a stronger neutrophilic and platelet-associated response, whereas Marchigiana maintained higher RBC and Hb values under HS. These observations suggest, but do not directly demonstrate, a more balanced systemic response in the Marchigiana population.

The contrast between Marchigiana and Holstein was particularly informative in defining the biological signature potentially associated with adaptive response to HS (Table 1). Compared with Holstein, Marchigiana showed higher expression of genes linked to inhibitory or tolerance-associated immune regulation, including *BTLA* (B and T lymphocyte associated), *THEMIS* (thymocyte selection associated), *DTX1* (deltex E3 ubiquitin ligase 1), (66), *RNF128* (ring finger protein 128) (67), and *SIGLEC11* (sialic acid binding Ig like lectin 11) (68). This set of genes is compatible with a response in which immune activation is not suppressed, but more tightly controlled. In particular, *THEMIS* has been shown to modulate the signaling threshold of the inhibitory receptor *BTLA*, supporting the idea that Marchigiana may better restrain excessive immune escalation during thermal challenge (69).

In parallel, Marchigiana also showed enrichment of up-regulated genes involved in energy sensing, IGF-axis modulation, endothelial lipid handling, and erythroid/heme homeostasis, such as *PRKAA2* (protein kinase AMP-activated catalytic subunit alpha 2), *IGFBP4* (insulin like growth factor binding protein 4), *GPIHBP1* (glycosylphosphatidylinositol anchored high density lipoprotein binding protein 1), and *ALAS2* (5′-aminolevulinate synthase 2). This interpretation is biologically plausible because AMPK, whose catalytic α2 subunit is encoded by *PRKAA2*, is a central cellular energy sensor and has also been implicated in metabolic adaptation to HS in dairy cattle (70, 71). *IGFBP4* is better interpreted here as a modulator of local IGF bioavailability, with possible implications for adipose and vascular homeostasis, rather than as a direct metabolic sensor (72, 73). In turn, *GPIHBP1* plays a key role in intravascular triglyceride metabolism by binding and transporting lipoprotein lipase to the capillary lumen, thus supporting the concept of more efficient endothelial lipid handling (74). Finally, *ALAS2* encodes the erythroid-specific rate-limiting enzyme of heme biosynthesis, making it a plausible marker of erythroid/heme homeostasis and, in our study, a potentially relevant molecular correlate of the higher RBC and Hb values observed in Marchigiana under HS conditions (75, 76). Additional highly upregulated genes in Marchigiana, such as *EDIL3* (EGF-like repeats and discoidin I-like domains 3), *SPARC* (secreted protein acidic and cysteine rich), *COL5A3* (collagen type V alpha 3 chain), and *MMP15* (matrix metallopeptidase 15), further support the interpretation of a response oriented toward endothelial stability, extracellular organization, and controlled tissue adaptation, rather than the broader inflammatory-vascular remodeling program observed in Holstein (77–79).

By contrast, the Holstein-enriched side of the comparison was characterized by a broader inflammatory, immunometabolic, and vascular-remodeling program. The higher expression of chemokines and cytokine-related genes, including *CXCL13* (C-X-C motif chemokine ligand 13), *CCL20* (C-C motif chemokine ligand 20), *CCL22* (C-C motif chemokine ligand 22), *CCL8* (C-C motif chemokine ligand 8), *CX3CL1* (C-X3-C motif chemokine ligand 1), and *CSF3* (colony stimulating factor 3), together with innate/myeloid activation markers such as *TREM1* (triggering receptor expressed on myeloid cells 1), *IL1RAP* (interleukin 1 receptor accessory protein), *FCGR1A* (Fc gamma receptor Ia), *CLEC5A* (C-type lectin domain family 5 member A), *OASL* (2′-5′-oligoadenylate synthetase-like), and *ISG15* (ISG15 ubiquitin-like modifier), suggests stronger inflammatory activation and leukocyte recruitment in Holstein (80, 81). Holstein also displayed a marked immunometabolic signature, as indicated by *CD36* (CD36 molecule), *FABP5* (fatty acid binding protein 5), *ACSL4* (acyl-CoA synthetase long chain family member 4), *CH25H* (cholesterol 25-hydroxylase), *ABCA1* (ATP binding cassette subfamily A member 1), *ABCG1* (ATP binding cassette subfamily G member 1), *APOE* (apolipoprotein E), *LPL* (lipoprotein lipase), *LIPG* (lipase G, endothelial type), and *CMKLR1* (chemerin chemokine-like receptor 1), consistent with lipid-driven immune activation and a higher metabolic cost of adaptation (82–84). In parallel, genes related to endothelial activation and extracellular matrix turnover, including *VEGFA* (vascular endothelial growth factor A), *VEGFC* (vascular endothelial growth factor C), *FLT1* (fms related receptor tyrosine kinase 1), *FLT4* (fms related receptor tyrosine kinase 4), *KDR* (kinase insert domain receptor), *PDPN* (podoplanin), *VCAM1* (vascular cell adhesion molecule 1), *VCAN* (versican), *COL4A1* (collagen type IV alpha 1 chain), *LAMA5* (laminin subunit alpha 5), *YAP1* (YAP1 transcriptional regulator), *WWTR1* (WW domain containing transcription regulator 1), *MMP1* (matrix metallopeptidase 1), *MMP12* (matrix metallopeptidase 12), *MMP14* (matrix metallopeptidase 14), and *ADAMTS1/4/8* (a disintegrin and metalloproteinase with thrombospondin motifs 1, 4, and 8) support a stronger vascular and extracellular-remodeling response (85–87). Finally, the enrichment of stress-responsive genes such as *HMOX1* (heme oxygenase 1) and *GDF15* (growth differentiation factor 15), suggests that this broader inflammatory burden was accompanied by compensatory cytoprotective and feedback-regulatory mechanisms (88, 89). Overall, these findings support the interpretation that Holstein responded to HS with a broader and biologically more costly inflammatory-immunometabolic and tissue-remodeling program than Marchigiana.

The comparison between Marchigiana and Limousine revealed a less explicit, but biologically coherent, divergence (Table 1). In this case, Marchigiana appeared to preferentially activate a response centered on immune modulation, redox protection, and metabolic buffering, rather than the more overt inflammatory and remodeling profile observed in Limousine. This interpretation is consistent with the enrichment in Marchigiana of up-regulated genes such as *CTLA4* (cytotoxic T-lymphocyte associated protein 4), together with *KIR3DL2* (killer cell immunoglobulin like receptor, three Ig domains and long cytoplasmic tail 2), *IL21* (interleukin 21), *OSM* (oncostatin M), *SIGLEC1* (sialic acid binding Ig like lectin 1), and *CCL24* (C-C motif chemokine ligand 24), suggesting an immune environment that remains active but more tightly coordinated. In particular, *CTLA4* is a central immune checkpoint involved in immune homeostasis (90, 91), whereas *IL21* is a pleiotropic cytokine acting on both innate and adaptive immunity, and *OSM* has been linked not only to inflammatory signaling but also to tissue repair and regeneration (92–94).

Additional Marchigiana-enriched genes, including *WWC1* (WW and C2 domain containing 1), *WNT5A* (Wnt family member 5A), *HEY2* (hes related family bHLH transcription factor with YRPW motif 2), *JUP* (junction plakoglobin), *GATA4* (GATA binding protein 4), and *LRG1* (leucine rich alpha-2-glycoprotein 1), further support the involvement of tissue integrity, mechanosensing, and controlled adaptive remodeling (95–97). By contrast, Limousine showed a more reactive inflammatory-remodeling profile, with higher expression of chemokine- and cytokine-related genes such as *CCL2* (C-C motif chemokine ligand 2), *CCL20* (C-C motif chemokine ligand 20), *CXCL3* (C-X-C motif chemokine ligand 3), *CSF2* (colony stimulating factor 2), *CSF3* (colony stimulating factor 3), *IL6* (interleukin 6), *IL9* (interleukin 9), *IL36A* (interleukin 36 alpha), together with *NOS2* (nitric oxide synthase 2), *PTGS2* (prostaglandin-endoperoxide synthase 2), *PDK4* (pyruvate dehydrogenase kinase 4), *RARRES2* (retinoic acid receptor responder 2), *MMP1* (matrix metallopeptidase 1), *PDPN* (podoplanin), *TFPI2* (tissue factor pathway inhibitor 2), *KDR* (kinase insert domain receptor), and *ADM* (adrenomedullin), consistent with a stronger inflammatory, immunometabolic, and vascular-remodeling response under HS (53, 98–101).

Overall, the integrated interpretation of the three population comparisons indicates that Marchigiana displayed transcriptional features consistent with a potentially more balanced adaptive response to HS under the investigated conditions. Previous genomic studies in Marchigiana cattle identified SNPs associated with resistance to bovine paratuberculosis, particularly involving genes related to immune regulation and host defense (14). Although these findings concern a different biological context, they support further investigation of whether Marchigiana cattle retain genetic architectures contributing to robustness under environmental and physiological challenges. The transcriptional profile observed in Marchigiana was consistent with coordinated regulation of immune, redox, metabolic, and tissue-remodeling processes, suggesting a more balanced adaptive response to thermal challenge. By contrast, the stronger enrichment in Holstein and, to a lesser extent, in Limousine of genes related to chemokine recruitment, IL-1/TREM1-associated amplification, lipid-driven immune activation, nitric oxide/prostaglandin pathways, angiogenesis, and extracellular matrix turnover was consistent with a response that may be more energetically demanding and potentially more disruptive to homeostasis. These population-associated signatures highlight candidate pathways for future controlled, multi-farm studies designed to test physiological and productive resilience directly.

To complement the transcriptomic analysis, cis-eQTL mapping was included as a regulatory genomics layer aimed at linking inter-individual variation in blood gene expression to nearby genetic variants. This approach is particularly relevant in the context of heat stress because differential expression analysis identifies genes and pathways that respond to thermal challenge, whereas eQTL mapping can highlight genes whose expression is also influenced by cis-regulatory genetic variation. Therefore, the integration of RNA-seq and genotype data may help prioritize candidate genes that are not only transcriptionally responsive, but also genetically regulated and potentially more informative for future selection strategies (21–25).

Among the most significant signals, two cis-eQTLs with the lowest FDR regulated Epoxide Hydrolase 2 (*EPHX2)* in both the imputed and non-imputed datasets. *EPHX2* encodes a soluble epoxide hydrolase, an enzyme involved in the metabolism of bioactive lipid mediators that participate in vascular, inflammatory, and metabolic regulation (102). In the context of heat stress, where vascular adaptation, oxidative imbalance, inflammation, and energy metabolism are tightly interconnected, genetic regulation of *EPHX2* expression may be relevant for modulating individual differences in systemic adaptation. This finding is coherent with the broader transcriptomic evidence observed in this study, where breed-related differences involved inflammatory signaling, lipid-related immunometabolic pathways, and vascular remodeling.

The identification of cis-eQTL associated with granulosyn *(GNLY)* and Granzyme B (*GZMB)* further supports the involvement of genetically regulated immune effector pathways in response to thermal challenge. Both genes are linked to cytotoxic T-cell and natural killer cell activity and may therefore reflect regulatory variation affecting leukocyte-mediated immune responses (103–106). This is particularly relevant because the transcriptomic analyses showed that heat stress induced immune- and inflammation-related pathways across all breeds, but with different intensity and organization. In this framework, eQTL associated with cytotoxic and inflammatory genes may help explain part of the inter-individual variability in the magnitude or regulation of immune activation under heat-stress conditions.

Additional highly ranked eQTL target genes, including Heme-Binding Protein 2 (*HEBP2)* and Solute Carrier Family 38 Member 4 (*SLC38A4)*, suggest that regulatory variation may also involve pathways related to mitochondrial stress, cell-death regulation, amino acid transport, and nutrient sensing. These biological functions are consistent with the concept that heat-stress adaptation requires not only immune modulation, but also metabolic buffering and maintenance of cellular homeostasis. In particular, *SLC38A4*, as a member of the solute carrier family involved in amino acid transport and nutrient-sensing pathways, may be relevant in conditions where animals need to reprioritize energy and nutrient allocation to cope with environmental stress (107–109).

The direct integration of differential-expression and cis-eQTL results identified six transcripts showing both an HS-associated expression change and evidence of local genetic regulation. Among the protein-coding candidates, GPRC5B provides the clearest connection with the population-specific transcriptomic response: it was upregulated during HS in Holstein cattle and its cis-eQTL association met FDR ≤ 0.05. GPRC5B has been implicated in pro-inflammatory signaling in vascular cells (110) which is consistent with the stronger inflammatory and vascular-remodeling profile observed in this population. CHRND, encoding the delta subunit of the muscle nicotinic acetylcholine receptor, was downregulated in Holstein cattle and also showed evidence of local genetic regulation at FDR ≤ 0.05. CHRND has been proposed as a candidate gene associated with adaptive responses to environmental stress in sheep (111). However, because expression was measured in blood and no neuromuscular phenotype was assessed, it's possible contribution to systemic heat-stress adaptation remains uncertain. KRTAP11-1 showed the largest positive expression change, but its cis-eQTL association met only the exploratory FDR ≤ 0.10 threshold; moreover, its known relationship with keratin-associated structures and skin adaptation in studies investigating environmental responses in livestock (112, 113) does not provide direct evidence of coat or skin remodeling in a blood-based dataset. NRL was upregulated in Marchigiana cattle, but its low expression and exploratory cis-eQTL association similarly warrant cautious interpretation.

Two currently unnamed lncRNA genes were also identified. ENSBTAG00000060972 was upregulated in Limousine cattle and associated with a cis-eQTL at FDR ≤ 0.05, whereas ENSBTAG00000063123 was upregulated in Holstein cattle and met the exploratory FDR ≤ 0.10 threshold. Previous studies have demonstrated extensive changes in bovine lncRNA expression during heat stress and have suggested that non-coding transcripts may contribute to stress-responsive regulatory networks (114, 115). Nevertheless, the two lncRNAs identified here have no assigned gene symbol, annotated ortholog, or experimentally validated target genes. Their concurrent differential expression and cis-eQTL association therefore prioritize them as novel candidate regulatory transcripts, but do not establish a direct role in thermotolerance.

Importantly, cis-eQTL mapping was performed across HS and TN samples, with condition included as a covariate. Consequently, the detected associations indicate that overall inter-individual variation in the expression of these transcripts is partly associated with local genetic variation; they do not demonstrate that variants regulate expression specifically during HS. Formal genotype-by-environment interaction analyses, together with lncRNA–mRNA co-expression, target prediction, and functional validation, will be required to determine whether these candidates contribute to heat-stress adaptation.

Taken together, the transcriptomic and cis-eQTL findings indicate that heat-stress response involves both inducible molecular pathways and genetically regulated variation in gene expression. This supports the value of integrating molecular biomarkers with sensor-derived phenotypes, particularly in Marchigiana cattle, where wearable devices allowed the exploration of inter-individual variability under field heat-stress conditions.

In this context, the sensor-derived data collected in Marchigiana cattle provided a complementary phenotypic layer for exploring inter-individual variability under field heat-stress conditions. The unsupervised clustering analysis based on movement intensity and ΔTHI profiles identified a small subgroup of animals characterized by partially distinct behavioral-environmental patterns, which also tended to show a more coherent transcriptomic distribution. Although these exploratory clusters should not be interpreted as fixed biological phenotypes, the partial convergence between sensor-derived profiles and blood transcriptomic signatures suggests that wearable sensors may capture biologically meaningful variability among animals exposed to the same environmental challenge. This pattern may indicate that a subset of animals responded to HS through a more distinct adaptive strategy involving differences in activity dynamics, animal environmental exposure, and inflammatory signaling pathways, including MAPK-related responses. This observation is consistent with previous transcriptomic evidence in cattle showing that chronic HS can induce the upregulation of MAPK-related signaling pathways, together with other stress- and inflammation-associated responses (110). The possible involvement of inflammatory and stress-related pathways, including MAPK-related signaling, further suggests that behavioral-environmental variability detected by PLF tools may reflect, at least in part, differences in the biological organization of the heat-stress response. Overall, these findings support the value of integrating PLF-derived phenotypes with molecular and regulatory biomarkers to better identify animals with different adaptive strategies under heat stress.

Several limitations should be considered when interpreting these findings. First, each breed was represented by animals from a different farm; consequently, breed was intrinsically confounded with farm-specific factors, including management, diet, parasite exposure, physiological and productive status, and local microclimatic conditions. Although all populations were managed under semi-extensive conditions and HS and TN periods were defined using THI thresholds, the observed contrasts cannot be attributed exclusively to breed genetics. Second, wearable sensors were available only for Marchigiana cattle. The PLF component was therefore designed solely as an exploratory within-population analysis of inter-individual variability and cannot support direct behavioral comparisons among breeds. Third, restraint and venipuncture are acute stressors that may induce short-term hematological, immunological, and transcriptomic changes. However, the sampling protocol was standardized between the HS and TN sessions: animals were sampled at approximately 06:30 h, when they approached the feed bunk after feed delivery and entered the headlocks; the same immobilization and venipuncture procedures were used, and blood collection was completed within a short and comparable interval. The duration of restraint is relevant because handling-related cortisol changes can depend strongly on the interval between immobilization and sampling (116). Therefore, although an acute effect of handling cannot be completely excluded, the standardized procedure makes a systematic difference in pre-sampling stress between the HS and TN sessions less likely. Lastly, the sensor-derived clustering should also be interpreted with caution, since Cluster 3 included only three animals. Therefore, the clustering analysis was considered exploratory and hypothesis-generating, rather than confirmatory, and was used only to describe within-Marchigiana inter-individual variability under heat stress.

## 5 Conclusion

These findings indicate that the three cattle populations exhibited distinct hematological and transcriptomic responses under the investigated HS conditions. Because each breed was sampled from a different farm, the observed differences cannot be attributed exclusively to genetic differences among breeds and should instead be interpreted as breed-associated patterns potentially influenced by farm-specific environmental and management factor. Holstein cattle showed a more extensive inflammatory, immunometabolic, and tissue-remodeling response, potentially associated with greater biological costs, whereas Limousine displayed an intermediate reactive profile. Marchigiana cattle exhibited a more coordinated pattern involving immune regulation, redox protection, metabolic buffering, and less pronounced extracellular matrix remodeling. These features are consistent with a more balanced adaptive response to thermal challenge. Since productive performance and direct physiological thermotolerance traits were not measured, the present results should be considered candidate signatures of adaptive capacity rather than definitive evidence of greater resilience.

The inclusion of cis-eQTL mapping provided an additional regulatory layer to the transcriptomic analysis, highlighting genes whose expression may be influenced by nearby genetic variants. Although these results are exploratory and require validation in larger independent cohorts and through genotype-by-environment analyses, they prioritize candidate regulatory markers for future studies of heat adaptation. The IoT collars used in Marchigiana cattle provided useful field-based information on behavioral and animal-proximal environmental variation, supporting the value of PLF as a complementary within-population phenotyping tool. Future integration of regulatory genomics, standardized physiological measurements, productive traits, and PLF-derived phenotypes across breeds and farms may support more robust strategies to improve adaptation, health, welfare, and sustainability in livestock populations.

## Data Availability

The datasets presented in this study can be found in online repositories. The names of the repository/repositories and accession number(s) can be found below: NCBI Sequence Read Archive (SRA), BioProject accession number PRJNA1472634 (BioSamples SAMN60508669 to SAMN60508782).
