# Supplementary material for: Precision livestock farming and integrated molecular profiling reveal heat-stress resilience signatures in Marchigiana cattle
Source: Front Vet Sci. 2026 Sep 17;13:1895058. doi: 10.3389/fvets.2026.1895058 (PMC13629503; doi:10.3389/fvets.2026.1895058)
Supplement: Supplementary file 1 [file Data_Sheet_1.zip › Supplementary/Supplementary_Figures.docx]

**Figure S1**. Principal Component Analysis (PCA) performed on movement and THI data recorded by sensors for 16 Marchigiana cattle during the heat stress (HS) period. Point colors indicate the clusters identified through KNN classification.

**Figure S2**. Analysis of ΔTHI and VM values for individual animals. The x-axis represents the activity hour. Individual points within the boxplots represent hourly measurements collected for each animal during the HS period. The colors of the boxplots and points reflect animal classification according to the KNN-derived clusters (1, 2, or 3). Significance bars indicate statistically significant differences identified through pairwise Wilcoxon–Mann–Whitney tests with Bonferroni correction for multiple comparisons.

**Figure S3**. PCA performed on transcriptomic data from Marchigiana cattle during the HS period. Point colors reflect animal classification according to the KNN-derived clusters (1, 2, or 3).

**Figure S4**. Volcano plot analysis of differential gene expression in Marchigiana cattle during the HS period. Animals were labeled according to the clusters identified through KNN analysis of sensor-derived data. Since three clusters were identified, three pairwise comparisons are shown: 1) cluster 1 vs. cluster 2, 2) cluster 1 vs. cluster 3, and 3) cluster 2 vs. cluster 3. DEG colors reflect the KNN-based animal classification (1, 2, or 3).

**Figure S5**. Differential abundance analysis performed on the KEGG database using the DEGs identified in comparisons between Marchigiana cattle belonging to: A) cluster 1 vs. cluster 3 and B) cluster 2 vs. cluster 3.
