# Supplementary material for: Precision livestock farming and integrated molecular profiling reveal heat-stress resilience signatures in Marchigiana cattle
Source: Front Vet Sci. 2026 Sep 17;13:1895058. doi: 10.3389/fvets.2026.1895058 (PMC13629503; doi:10.3389/fvets.2026.1895058)
Supplement: Supplementary file 1 [file Data_Sheet_1.zip › Supplementary/Supplementary_table_1_stats.docx]

| Supplementary Table 1. Sequencing statistics. | | | | | |
| --- | --- | --- | --- | --- | --- |
| Library_ID | Breed | Period | Raw Reads (M) | Cleaned Reads | Uniquelly mapped Reads |
| 1-F2 | Holstein | HS | 90.4 | 44,390,261 | 42,942,745 |
| 2-F2 | Holstein | TN | 87.64 | 43,400,489 | 42,411,943 |
| 3-F5 | Holstein | HS | 78.16 | 38,694,420 | 37,703,966 |
| 4-F5 | Holstein | TN | 77.47 | 38,432,671 | 37,491,037 |
| 5-F6 | Holstein | HS | 75.63 | 37,508,415 | 36,452,996 |
| 6-F6 | Holstein | TN | 85.23 | 42,281,165 | 41,165,268 |
| 7-F8 | Holstein | HS | 97.75 | 48,449,800 | 47,300,047 |
| 8-F8 | Holstein | TN | 87.66 | 43,391,613 | 42,291,371 |
| 9-F10 | Holstein | HS | 83.79 | 41,367,627 | 40,094,904 |
| 10-F10 | Holstein | TN | 81.76 | 40,530,874 | 39,489,569 |
| 11-F12 | Holstein | HS | 75.79 | 37,538,218 | 36,604,318 |
| 12-F12 | Holstein | TN | 78.88 | 39,084,429 | 38,083,309 |
| 13-F17 | Holstein | HS | 79.24 | 39,127,922 | 38,089,058 |
| 14-F17 | Holstein | TN | 75.95 | 37,658,934 | 36,482,466 |
| 15-F19 | Holstein | HS | 93.4 | 46,379,198 | 45,272,491 |
| 16-F19 | Holstein | TN | 75.35 | 37,386,296 | 36,428,881 |
| 17-F25 | Holstein | HS | 81.61 | 40,240,247 | 38,960,236 |
| 18-F25 | Holstein | TN | 74.55 | 36,944,948 | 35,989,997 |
| 19-M1 | Marchigiana | HS | 73.95 | 36,586,579 | 35,681,853 |
| 20-M1 | Marchigiana | TN | 83.48 | 41,335,088 | 40,267,151 |
| 21-M2 | Marchigiana | HS | 79.11 | 38,971,535 | 37,967,858 |
| 22-M2 | Marchigiana | TN | 77.04 | 38,082,310 | 37,071,439 |
| 23-M3 | Marchigiana | HS | 81.5 | 40,292,051 | 39,353,413 |
| 24-M3 | Marchigiana | TN | 71.05 | 35,171,234 | 34,287,241 |
| 25-M4 | Marchigiana | HS | 75.32 | 37,103,412 | 35,906,513 |
| 26-M4 | Marchigiana | TN | 72.79 | 36,092,562 | 35,141,126 |
| 27-M5 | Marchigiana | HS | 69.73 | 34,527,852 | 33,626,621 |
| 28-M5 | Marchigiana | TN | 80.15 | 39,641,519 | 38,607,260 |
| 29-M6 | Marchigiana | HS | 74.26 | 36,898,389 | 35,965,806 |
| 30-M6 | Marchigiana | TN | 84.05 | 41,690,723 | 40,713,343 |
| 31-M7 | Marchigiana | HS | 83.94 | 41,458,377 | 40,446,210 |
| 32-M7 | Marchigiana | TN | 83.39 | 41,364,818 | 40,430,341 |
| 33-M8 | Marchigiana | HS | 74.75 | 37,005,542 | 35,774,017 |
| 34-M8 | Marchigiana | TN | 72.91 | 36,096,505 | 35,125,101 |
| 35-M9 | Marchigiana | HS | 79.81 | 39,443,456 | 38,590,082 |
| 36-M9 | Marchigiana | TN | 73.6 | 36,393,699 | 35,371,457 |
| 37-M10 | Marchigiana | HS | 98.18 | 47,707,496 | 46,392,378 |
| 38-M10 | Marchigiana | TN | 75.09 | 37,209,908 | 36,331,005 |
| 39-M11 | Marchigiana | HS | 74.68 | 36,882,655 | 35,987,948 |
| 40-M11 | Marchigiana | TN | 71.46 | 35,233,268 | 34,322,731 |
| 41-M12 | Marchigiana | HS | 89.57 | 44,103,119 | 42,862,045 |
| 42-M12 | Marchigiana | TN | 71.16 | 35,200,676 | 34,310,696 |
| 43-M13 | Marchigiana | HS | 74.9 | 36,993,445 | 36,140,926 |
| 44-M13 | Marchigiana | TN | 77.34 | 37,749,605 | 36,708,662 |
| 45-M14 | Marchigiana | HS | 66.7 | 32,907,286 | 31,915,995 |
| 46-M14 | Marchigiana | TN | 66.23 | 32,781,428 | 31,951,210 |
| 47-M15 | Marchigiana | HS | 69.55 | 34,420,533 | 33,476,962 |
| 48-M15 | Marchigiana | TN | 68.25 | 33,753,667 | 32,858,339 |
| 49-M16 | Marchigiana | HS | 67.22 | 33,255,534 | 32,284,377 |
| 50-M16 | Marchigiana | TN | 84.22 | 41,661,116 | 40,538,235 |
| 51-M17 | Marchigiana | HS | 69.35 | 34,291,763 | 33,435,618 |
| 52-M17 | Marchigiana | TN | 84.26 | 41,669,333 | 40,561,013 |
| 53-M18 | Marchigiana | HS | 65.84 | 31,620,057 | 30,675,499 |
| 54-M18 | Marchigiana | TN | 82.9 | 40,980,801 | 39,967,112 |
| 55-M19 | Marchigiana | HS | 71.16 | 34,962,067 | 34,037,905 |
| 56-M19 | Marchigiana | TN | 74.44 | 36,923,485 | 36,010,211 |
| 57-M20 | Marchigiana | HS | 71.6 | 35,454,630 | 34,543,895 |
| 58-M20 | Marchigiana | TN | 69.2 | 34,226,707 | 33,281,729 |
| 59-M21 | Marchigiana | HS | 77.1 | 38,213,886 | 37,343,452 |
| 60-M21 | Marchigiana | TN | 67.47 | 33,413,184 | 32,566,203 |
| 61-M22 | Marchigiana | HS | 66.43 | 32,881,484 | 32,075,502 |
| 62-M22 | Marchigiana | TN | 69.05 | 34,211,881 | 33,301,435 |
| 63-M23 | Marchigiana | HS | 67.33 | 33,331,846 | 32,612,059 |
| 64-M23 | Marchigiana | TN | 73.04 | 36,206,474 | 35,361,613 |
| 65-M24 | Marchigiana | HS | 93.42 | 46,241,282 | 44,960,553 |
| 66-M24 | Marchigiana | TN | 88.53 | 43,859,622 | 42,603,074 |
| 67-M25 | Marchigiana | HS | 82.56 | 40,768,652 | 39,724,502 |
| 68-M25 | Marchigiana | TN | 72.95 | 36,101,584 | 35,084,054 |
| 69-M26 | Marchigiana | HS | 75.25 | 36,974,967 | 36,080,211 |
| 70-M26 | Marchigiana | TN | 69.75 | 34,507,079 | 33,660,865 |
| 71-M27 | Marchigiana | HS | 79.04 | 39,016,982 | 37,951,141 |
| 72-M27 | Marchigiana | TN | 77.76 | 38,348,954 | 37,363,901 |
| 73-M29 | Marchigiana | HS | 69.89 | 34,547,021 | 33,522,554 |
| 74-M29 | Marchigiana | TN | 86.29 | 42,736,303 | 41,568,359 |
| 75-L1 | Limousine | HS | 97.28 | 48,172,987 | 47,226,328 |
| 76-L1 | Limousine | TN | 63.28 | 31,341,092 | 30,592,715 |
| 77-L3 | Limousine | HS | 75.02 | 37,083,985 | 36,265,006 |
| 78-L3 | Limousine | TN | 73.66 | 36,467,666 | 35,670,326 |
| 79-L4 | Limousine | HS | 84.66 | 41,775,869 | 40,769,915 |
| 80-L4 | Limousine | TN | 74.14 | 36,666,708 | 35,718,521 |
| 81-L6 | Limousine | HS | 72.48 | 35,905,042 | 34,849,986 |
| 82-L6 | Limousine | TN | 81.72 | 40,331,150 | 39,326,372 |
| 83-L7 | Limousine | HS | 93.18 | 45,937,014 | 44,740,987 |
| 84-L7 | Limousine | TN | 67.8 | 33,507,663 | 32,730,067 |
| 85-L9 | Limousine | HS | 60.69 | 29,883,242 | 29,083,910 |
| 86-L9 | Limousine | TN | 68.41 | 33,812,622 | 32,922,820 |
| 87-L11 | Limousine | HS | 84.21 | 41,425,567 | 40,269,932 |
| 88-L11 | Limousine | TN | 73.24 | 36,203,041 | 35,128,459 |
| 89-L12 | Limousine | HS | 65.17 | 32,199,345 | 30,773,970 |
| 90-L12 | Limousine | TN | 68.26 | 33,836,456 | 32,747,707 |
| 91-L13 | Limousine | HS | 66.63 | 32,997,357 | 31,721,260 |
| 92-L13 | Limousine | TN | 74.4 | 36,800,485 | 35,067,597 |
| 93-L16 | Limousine | HS | 79.56 | 39,428,465 | 37,898,628 |
| 94-L16 | Limousine | TN | 72.47 | 35,884,336 | 34,548,565 |
| 95-L17 | Limousine | HS | 101.67 | 49,725,621 | 47,605,715 |
| 96-L17 | Limousine | TN | 84.25 | 41,770,510 | 40,708,388 |
| 97-L18 | Limousine | HS | 66.34 | 32,875,715 | 31,660,858 |
| 98-L18 | Limousine | TN | 68.64 | 34,025,597 | 33,020,122 |
| 99-L19 | Limousine | HS | 57.45 | 28,496,679 | 27,654,751 |
| 100-L19 | Limousine | TN | 58.55 | 28,942,531 | 27,962,537 |
| 101-L20 | Limousine | HS | 82.86 | 41,041,829 | 39,585,180 |
| 102-L20 | Limousine | TN | 61.16 | 30,325,939 | 29,549,799 |
| 103-L22 | Limousine | HS | 61.63 | 30,179,570 | 28,975,319 |
| 104-L22 | Limousine | TN | 68.31 | 33,871,686 | 33,019,229 |
| 105-L23 | Limousine | HS | 57.24 | 28,353,851 | 27,308,500 |
| 106-L23 | Limousine | TN | 57.49 | 28,463,229 | 27,615,961 |
| 107-L24 | Limousine | HS | 98.66 | 48,716,254 | 47,007,451 |
| 108-L24 | Limousine | TN | 68.04 | 33,612,901 | 32,556,086 |
| 109-L25 | Limousine | HS | 80.08 | 39,357,387 | 37,858,494 |
| 110-L25 | Limousine | TN | 93.26 | 46,087,906 | 44,943,440 |
| 111-L26 | Limousine | HS | 61.87 | 30,399,557 | 29,268,951 |
| 112-L26 | Limousine | TN | 68.9 | 34,146,521 | 33,187,416 |
| 113-L27 | Limousine | HS | 58.46 | 28,992,708 | 27,920,952 |
| 114-L27 | Limousine | TN | 57.51 | 28,479,735 | 27,610,608 |
| Average |  |  | 75.78 | 37,454,726.11 | 36,412,528.34 |
